# Dependence of column ozone on future ODSs and GHGs in the variability of 500-ensemble members

**DOI:** 10.1038/s41598-023-27635-y

**Published:** 2023-01-06

**Authors:** Hideharu Akiyoshi, Masanao Kadowaki, Yousuke Yamashita, Toshiharu Nagatomo

**Affiliations:** 1 Earth System Division, National Institute for Environmental Studies, 16-2 Onogawa, Tsukuba, Ibaraki 305-8506 Japan; 2 Nuclear Science and Engineering Center, Japan Atomic Energy Agency, 765-1 Funaishikawa, Tokai-mura, Naka-gun, Ibaraki 319-1184 Japan; 3 Japan Agency for Marine-Earth Science and Technology, 3173-25 Showa-machi, Kanazawa-ku, Yokohama, Kanagawa 236-0001 Japan

**Keywords:** Atmospheric chemistry, Atmospheric chemistry, Atmospheric dynamics

## Abstract

State-of-the-art chemistry–climate models (CCMs) have indicated that a future decrease in ozone-depleting substances (ODSs) combined with an increase in greenhouse gases (GHGs) would increase the column ozone amount in most regions except the tropics and Antarctic. However, large Arctic ozone losses have occurred at a frequency of approximately once per decade since the 1990s (1997, 2011 and 2020), despite the ODS concentration peaking in the mid-1990s. To understand this, CCMs were used to conduct 24 experiments with ODS and GHG concentrations set based on predicted values for future years; each experiment consisted of 500-member ensembles. The 50 ensemble members with the lowest column ozone in the mid- and high latitudes of the Northern Hemisphere showed a clear ODS dependence associated with low temperatures and a strong westerly zonal mean zonal wind. Even with high GHG concentrations, several ensemble members showed extremely low spring column ozone in the Arctic when ODS concentration remained above the 1980–1985 level. Hence, ODS concentrations should be reduced to avoid large ozone losses in the presence of a stable Arctic polar vortex. The average of the lowest 50 members indicates that GHG increase towards the end of the twenty-first century will not cause worse Arctic ozone depletion.

## Introduction

From its worst state in the 1990s, the ozone layer has been gradually recovering due to ozone-depleting substance (ODS)-reducing measures implemented internationally as a consequence of the Montreal Protocol and its amendments^1,2^. Ozone amounts and ozone recovery are affected by ODSs, such as chlorofluorocarbons^3–7^, and by greenhouse gases (GHGs)^8–12^. Moreover, in the high latitudes of the northern hemisphere (NH), ozone amounts in winter and spring are greatly influenced by interannual variations of the atmosphere^13,14^. Record lows in the total ozone during the Arctic spring were observed in 1997, 2011 and 2020^15,16^. A future projection using ODS values specified by the World Meteorological Organization (WMO) predicted that by the 2060s, total ozone may still episodically drop to 50–100 DU below the corresponding long-term ensemble mean^17^. A GHG increase may cause severe ozone loss in the Arctic in the future^18,19^. The large interannual variations in ozone amount are due to interannual variations in temperature and circulation, and hence are influenced by the large interannual variations of planetary waves^20,21^, which are partly influenced by the Quasi-Biennial Oscillation (QBO), the 11-year solar cycle and the El Niño Southern Oscillation (ENSO)^22–30^, but more predominantly by the intrinsic variability associated with wave–mean flow interaction^31,32^. Given the chaotic nature of the atmosphere, as wave propagation and dissipation are sensitive to zonal-wind and temperature fields^33^, a small difference in these fields may result in a large difference in the dynamical state of the future atmosphere^34^. Temperature and atmospheric circulation changes influence the ozone layer through chemical reactions and ozone transport, and ozone changes in turn influence temperature and circulation through radiation processes in the atmosphere^32,35,36^. The large interannual variations may make it difficult to understand the effects of ODSs and GHGs on long-term ozone variations^37^. In future, GHG concentrations will increase further^38–42^, while ODS concentrations will decrease owing to regulation^1,2,43^. Therefore, in order to predict the future behaviour of the ozone layer, it is essential to understand the dependence of ozone amounts on ODS and GHG concentrations in the context of interannual atmospheric variations. For this purpose, we performed 500-member ensemble simulations using chemistry–climate models (CCMs) constructed on the Model for Interdisciplinary Research on Climate (MIROC) versions 3.2 and 5. The CCMs were developed at Japan’s National Institute for Environmental Studies (NIES) and the University of Tokyo. The dependences of column ozone, polar cap temperature, and zonal mean zonal wind on ODS and GHG concentrations were analysed.

## Results

### MIROC3.2 CCM

As stated in the Introduction, there is a need to examine ozone dependence on ODS and GHG concentrations in the NH mid- and high latitudes in the context of interannual atmospheric variations. However, in this study, interannual variations due to the QBO, 11-year solar cycle and ENSO (see ‘Input Data for CCM run’ in the Methods section) were ignored; instead, the focus was on those caused by the intrinsic variations of the wave–mean flow interaction and the chaotic nature of fluid dynamics. This CCM setup still produced large interannual variations (variability among the ensemble members) in ozone and other atmospheric parameters in the NH mid- and high latitudes.

Twenty-four experiments were performed using pairings of ODS and GHG levels for selected years (Table 1). In addition, an experiment was performed using atmospheric data from the year 2000 (ODS-2000&GHG-2000) for comparison. Each experiment is a 510-year timeslice simulation that was run continuously, where each year is assumed to be an independent realization, but the first 10 years were excluded from the analysis. The ODS concentrations from past years were selected to simulate concentrations predicted to be achieved in the future under ODS regulation. For the ODS-1960&GHG-2040 pairing, minimal ozone destruction was predicted due to the low halogen concentrations; therefore, to save computing resources, no run was performed for this pairing. Instead, we assumed that ODS and GHG dependence for this pairing could be interpolated using the ODS-1960&GHG-2030 and ODS-1960&GHG-2050 results.

**Table 1:** Reference years for the ODS and GHG concentrations used for CCM runs with 500-member ensembles.

GHG	ODS				
	1960 (after 2100)	1980 (2044)	1985 (2030)	1990 (2006)	1995
2095	○	○	○	○	○
2050	○	○	○	○	○
2040	—	○	○	○	○
2030	○	○	○	○	○
2000	○	○	○	○	○

Each ○ indicates a pairing used for a CCM run; — indicates a pairing not used for a run. Years in parentheses are the years after 2000 with the same EESC levels as those before 2000 in the top row based on the WMO-A1 scenario. The GHG concentration levels for the years in the left column are based on the RCP 6.0 scenario.

ODS concentration levels were expressed using historical data from the WMO-A1 scenario. ODS concentrations are expected to decrease in the future, having peaked around 1995, due to ODS regulations. In the WMO-A1 scenario, equivalent effective stratospheric chlorine (EESC) in the polar regions is calculated by adding 65 times the number of bromine atoms to the number of chlorine atoms^44^; under this scenario, ODS concentration in 1990 corresponded to that in 2006, that in 1985 corresponded to that in 2030, that in 1980 corresponded to that in 2044, and that in 1960 corresponded to no year of the current century. GHG (CO_2_, CH_4_, and N_2_O) concentration levels were also expressed by year, based on the RCP 6.0 scenario.

**Figure 1:**
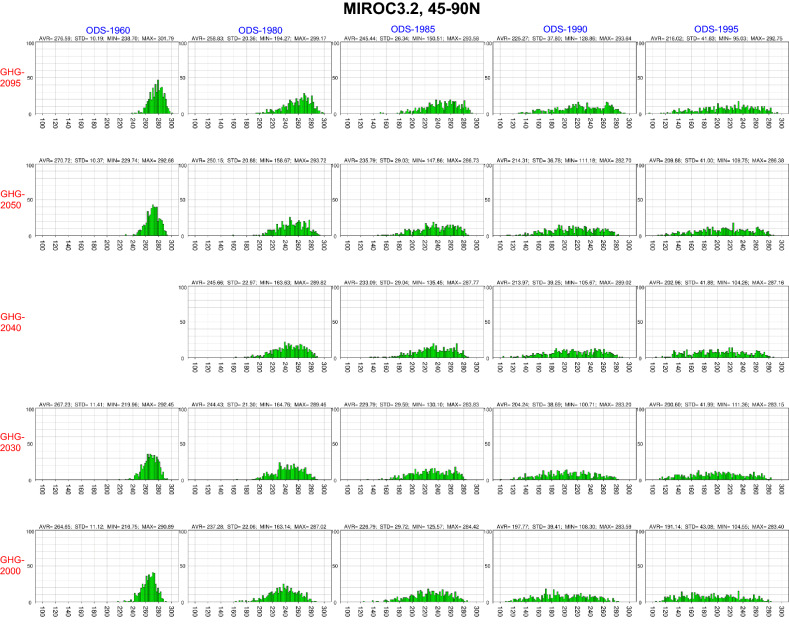
Histogram of the spring column ozone minimum values between 45° and 90° N from March to May for the 24 MIROC3.2 CCM runs with 500-member ensembles. Results are shown by panels for all runs using different ODS and GHG concentrations. The panels are arranged in the same order as in Table 1: the top row shows the GHG-2095 experiments, the bottom row the GHG-2000 experiments, the left column the ODS-1960 experiments, and the right column the ODS-1995 experiments. The spring column ozone minimum values are depicted on the horizontal axis at intervals of 20 DU. The size of each bin for the column ozone minima on the horizontal axis is 2 DU, with the bin of the smallest value at 90–92 DU and that of the largest value at 308–310 DU. The bin count (between 0 and 100) is indicated on the vertical axis. Column ozone values (average with standard deviation, maximum and minimum) for the 500-member ensembles of each experiment are represented in the upper parts of the panels.

Figure 1 shows histograms of the minimum column ozone values between 45° and 90° N from March to May for the 24 runs performed using the MIROC3.2 CCM, with the same arrangement of panels as in Table 1. At mid- and high latitudes during the NH spring, the distribution of minimum column ozone values was relatively narrow for the smallest ODS value (that for 1960 from the WMO-A1 scenario). As the ODS concentration increased (from left to right panels), the peak values of the distribution decreased. As can be seen in Fig. 1, the peak broadens and flattens, extending towards smaller minimum column ozone values. However, the largest minimum column ozone values remained relatively constant.

It is interesting to note that even under high-GHG conditions, several ensemble members exhibited very small column ozone minima in the presence of high ODS concentrations, such as the ODS-1995&GHG-2095 pairing (top right panel in Fig. 1). This suggests that extensive ozone depletion could occur in future high-GHG atmospheres if ODS concentrations remain high. For instance, Fig. 1 shows that the ensemble members with spring column ozone minima less than 200 DU notably increased with ODS concentrations above the 1985 level and with all selected GHG concentrations. Note that although the spring column ozone minimum is a good measure of the strength of spring ozone depletion in a region over a given time period, it may not comprehensively represent ozone depletion throughout the region and period, as it is a localized and instantaneous quantity. Thus, we also calculated the area from 45° to 90° N wherein the column ozone values were less than 220 DU from March to May (Supplementary Fig. S1) and the time-integrated value (Supplementary Fig. S2). The results showed that ensemble members with large ozone loss areas appeared when ODS concentrations were above those in 1985, which was consistent with the results of the analysis of the spring column ozone minimum. Hence, the spring column ozone minimum is an appropriate measure for detecting ensemble members showing extreme ozone depletion associated with changes in ODS concentration.

With increasing GHG concentrations (from bottom to top panels in Fig. 1), the peak of the distribution (maximum bin count) shifted to higher column ozone minima. This can be explained by an increase in ozone transport to high latitudes due to the strengthening of the meridional circulation when GHGs are more abundant^45–50^. The distribution of ozone loss areas (ozone values less than 220 DU) showed a trend consistent with that of the spring column ozone minima, with the area decreasing as GHGs increased (Supplementary Fig. S2).

**Figure 2:**
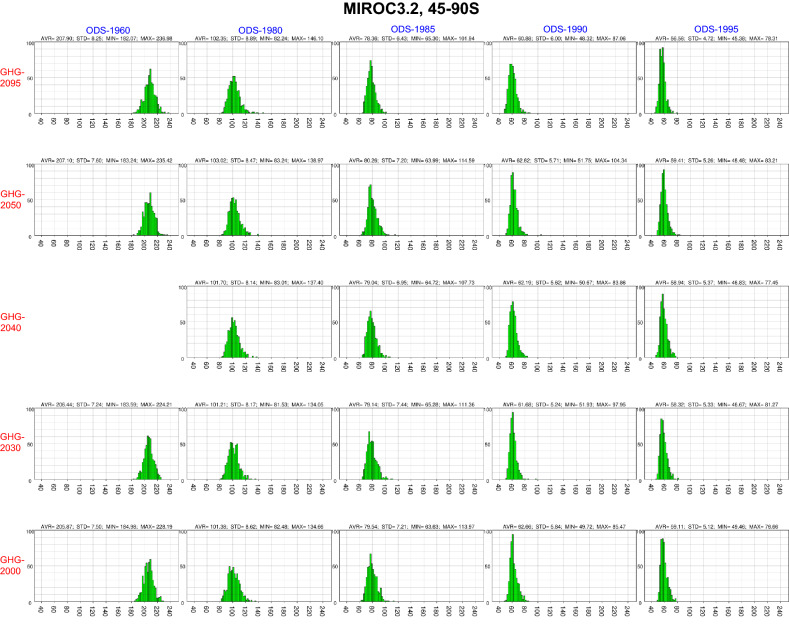
Histogram of the spring column ozone minimum values between 45° and 90° S from September to November for the 24 MIROC3.2 CCM runs with 500-member ensembles. Results are shown by panels for all runs using different ODS and GHG concentrations. The panels are arranged in the same order as in Table 1: the top row shows the GHG-2095 experiments, the bottom row the GHG-2000 experiments, the left column the ODS-1960 experiments, and the right column the ODS-1995 experiments. The spring column ozone minimum values are depicted on the horizontal axis at intervals of 20 DU. The bin size for the column ozone minima on the horizontal axis is 2 DU, with the bin of the smallest value at 30–32 DU and the largest at 248–250 DU. The bin count (between 0 and 100) is indicated on the vertical axis. Column ozone values (average with standard deviation, maximum and minimum) for the 500-member ensembles of each experiment are represented in the upper parts of the panels.

In the mid- and high latitudes during the Southern Hemisphere (SH) spring, the distributions were much narrower than those for the NH spring (Fig. 2). Increasing ODS concentrations (from left to right panels) induced a shift in the peak value towards lower column ozone minima and a narrower distribution. The narrowing of the column ozone minimum distribution implies less variability in that quantity among the ensemble members. The narrowing is considered to be a result from a nearly complete PSC-driven ozone destruction in the lower stratosphere, where most of the column ozone exists, at a grid and time indicating the column ozone minimum. Thus the column ozone minimum becomes less sensitive to meteorological variability as ODSs increase. The narrowing also may be associated with increased stability of the Antarctic polar vortex, with a stronger zonal mean zonal wind and with lower temperatures inside the vortex. However, narrowing of the distribution was not evident with regard to the area less than 220 DU as ODSs increase (Supplementary Fig. S3).

In contrast to the NH results, an increase in GHG concentration had little impact on the width and amplitude of the distribution of SH column ozone minima (from bottom to top panels in Fig. 2). The ozone loss area distribution also showed little dependence on the GHG concentration (Supplementary Fig. S3).

**Figure 3:**
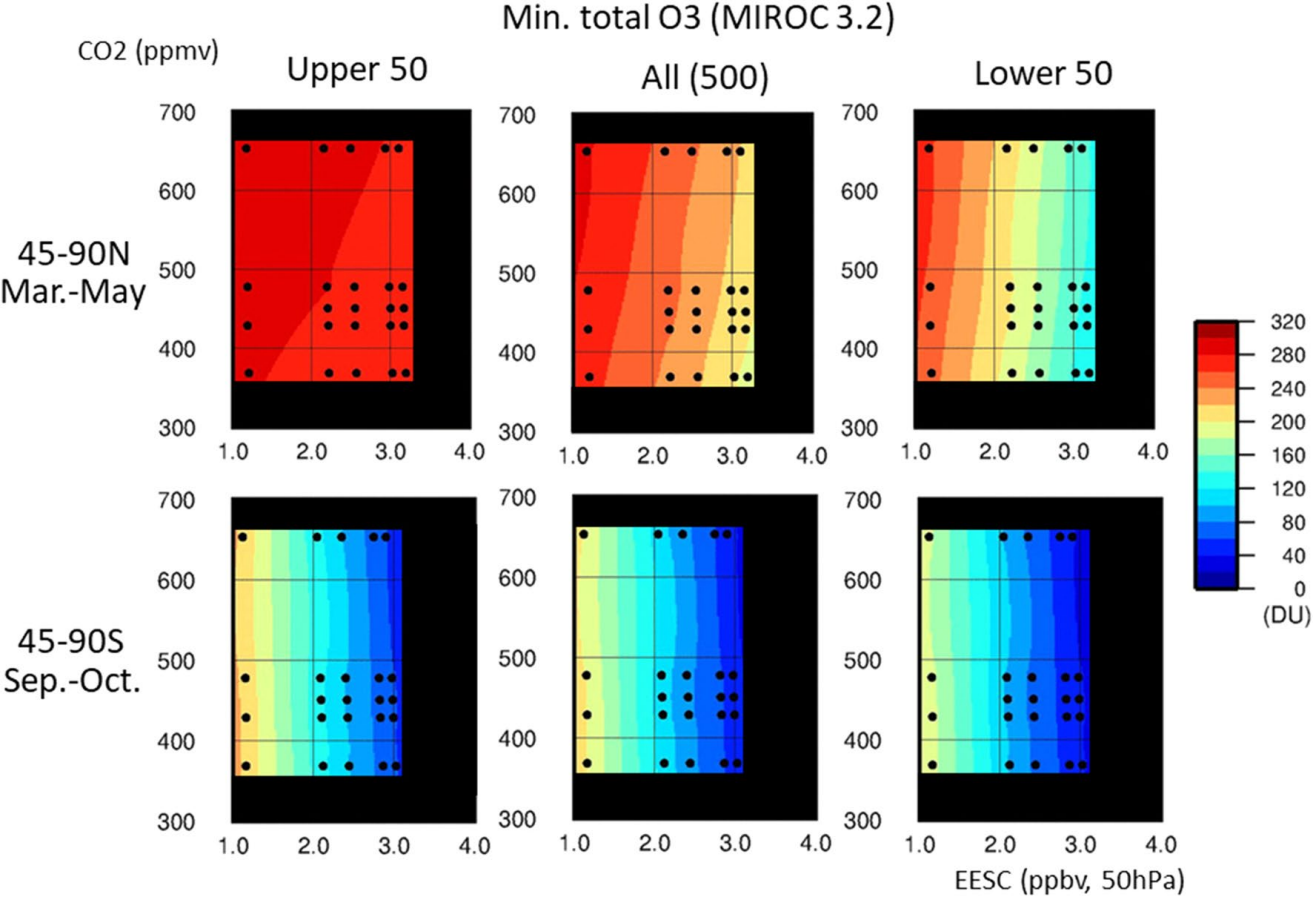
MIROC3.2 CCM results for the lower 50 ensemble members, full 500 members and upper 50 members regarding dependence of spring column ozone minimum values in the mid- and high latitudes on ODS and GHG concentrations. The upper panels show results for the area between 45° and 90° N from March to May (NH spring), and the lower panels show results for the area between 45° and 90° S from September to November (SH spring). Column ozone minimum values are averaged over the 50 ensemble members with the largest column ozone minima (upper 50, left), the full ensemble (500 members, middle) and the 50 members with the smallest column ozone minima (lower 50, right). The horizontal axis shows the mean spring EESC concentrations of the ODSs in ppbv for the NH (45–90° N, 50 hPa, March–May) and SH (45–90° S, 50 hPa, September–November). The vertical axis denotes the CO_2_ concentration in ppmv. Black circles denote the EESC and CO_2_ concentrations used in the 24 CCM runs. Colour levels represent column ozone minimum values in DU. The column ozone minimum values obtained from the 24 runs are interpolated and extrapolated in the (EESC, CO_2_) space. Most of the extrapolated regions are masked with black.

The NH mid- and high-latitude histograms (Fig. 1) suggest that the ODS and GHG dependence of the spring column ozone minimum differed between the 500-member ensemble mean and the means of the members with the most extreme column ozone values. Figure 3 shows the dependence on ODS and GHG concentrations of the ensemble-mean spring column ozone minima at mid- and high latitudes of both hemispheres, as predicted by the MIROC3.2 CCM. The 500-member ensemble mean is represented in the middle panels, the mean of the 50 members with the largest column ozone minimum (upper 50) is shown in the left panels, and the mean of the 50 members with the smallest column ozone minimum (lower 50) is shown in the right panels. The upper and lower 50 members represent extreme cases and statistically once-in-a-decade events, respectively. This grouping is based on the fact that Arctic springs with extremely low total ozone and chemical ozone losses tend to occur approximately once a decade (1997, 2011 and 2020)^15,16,51–59^.

In the NH mid- and high latitudes (upper panels), the mean spring column ozone minimum of the 500-member ensemble depended on both GHG levels (expressed on the vertical axis as CO_2_ concentrations) and ODS concentrations (expressed on the horizontal axis as EESC at 45°–90° N/S and 50 hPa during spring). The column ozone minima decreased as the ODS concentration increased, but increased with increasing GHG concentrations. For the upper 50 members, a very small dependence on ODS and GHG concentrations was evident. The average for the lower 50 members indicated clear ODS dependence.

**Figure 4:**
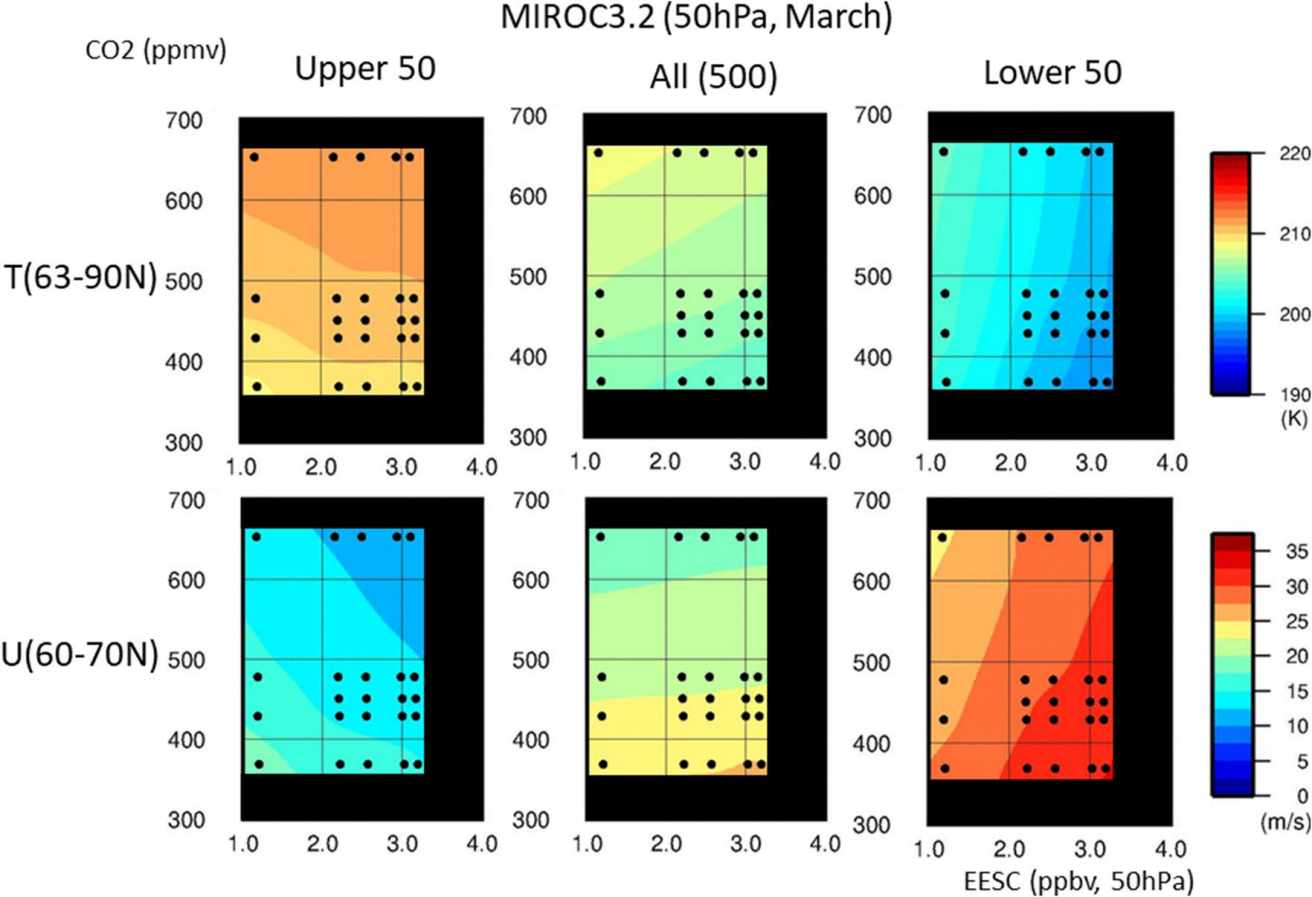
MIROC3.2 CCM results for the lower 50 ensemble members, full 500 members and upper 50 members regarding dependence of the polar cap temperature and polar night jet strength in the mid- and high latitudes of the NH (50 hPa, March) on ODS and GHG concentrations. The polar cap temperatures and polar night jet strength were calculated using the March mean values for daily temperature (63–90° N, 50 hPa) and daily zonal mean zonal wind speed (60–70° N, 50 hPa). Colour levels represent the temperature (in K) and zonal mean zonal wind speed (in m/s). The values of all experiments are interpolated and extrapolated in the (EESC, CO_2_) space. Most of the extrapolated regions are masked with black.

The difference between the means of the upper 50 and lower 50 members in the NH was likely caused by the large interannual variability of the Arctic polar vortex among the ensemble members. In the NH, catalytic destruction of ozone by ODSs appeared to occur at a significant level for ensemble members with a stable, colder polar vortex, whereas it did not develop under an unstable polar vortex. To confirm this, we examined the ODS and GHG dependence of the March values for the Arctic polar cap temperature at 63–90° N and 50 hPa and for the zonal mean zonal wind (polar night jet) at 60–70° N and 50 hPa, as shown in Fig. 4. The lower 50 ensemble members showed a clear ODS dependence. With increasing ODS concentrations, the anomalies became larger for both the polar cap temperature (colder) and polar night jet (stronger). This implies that for the lower 50 members, the Arctic polar vortex became stronger when the ODS concentration increased. The 500-member ensemble showed weak ODS dependence for the polar cap temperature and the polar night jet. For the upper 50 members, the polar cap temperature and polar night jet strength showed an ODS dependence trend opposite to that for the lower 50. For all three sets of members, the polar cap temperature increased and the polar night jet strength decreased with increasing GHG concentration.

However, the aforementioned results regarding ODS and GHG dependence of the polar night jet and polar cap temperature apply only to the lower stratosphere in the given latitudes, thus providing only a partial picture of the ODS and GHG dependence over the entire meridional section. The future decrease in ODS from the 1995 level to the 1960 level increases the polar cap temperature not only at 50 hPa but also in the entire polar stratosphere, as well as weakening the polar night jet in the stratosphere and mesosphere. The GHG increase from the 2000 level to the 2095 level also weakens the polar night jet by a smaller magnitude, but the temperature increase in the polar region was limited to the lower stratosphere (see Supplementary Figs. S4 and S6). Both the decreasing ODS and increasing GHG were associated with statistically significant differences (significance level 95% or more) among the lower 50 members with regard to polar cap temperature (63–90° N, 50 hPa) and polar night jet strength (60–70° N, 50 hPa) for March.

**Figure 5:**
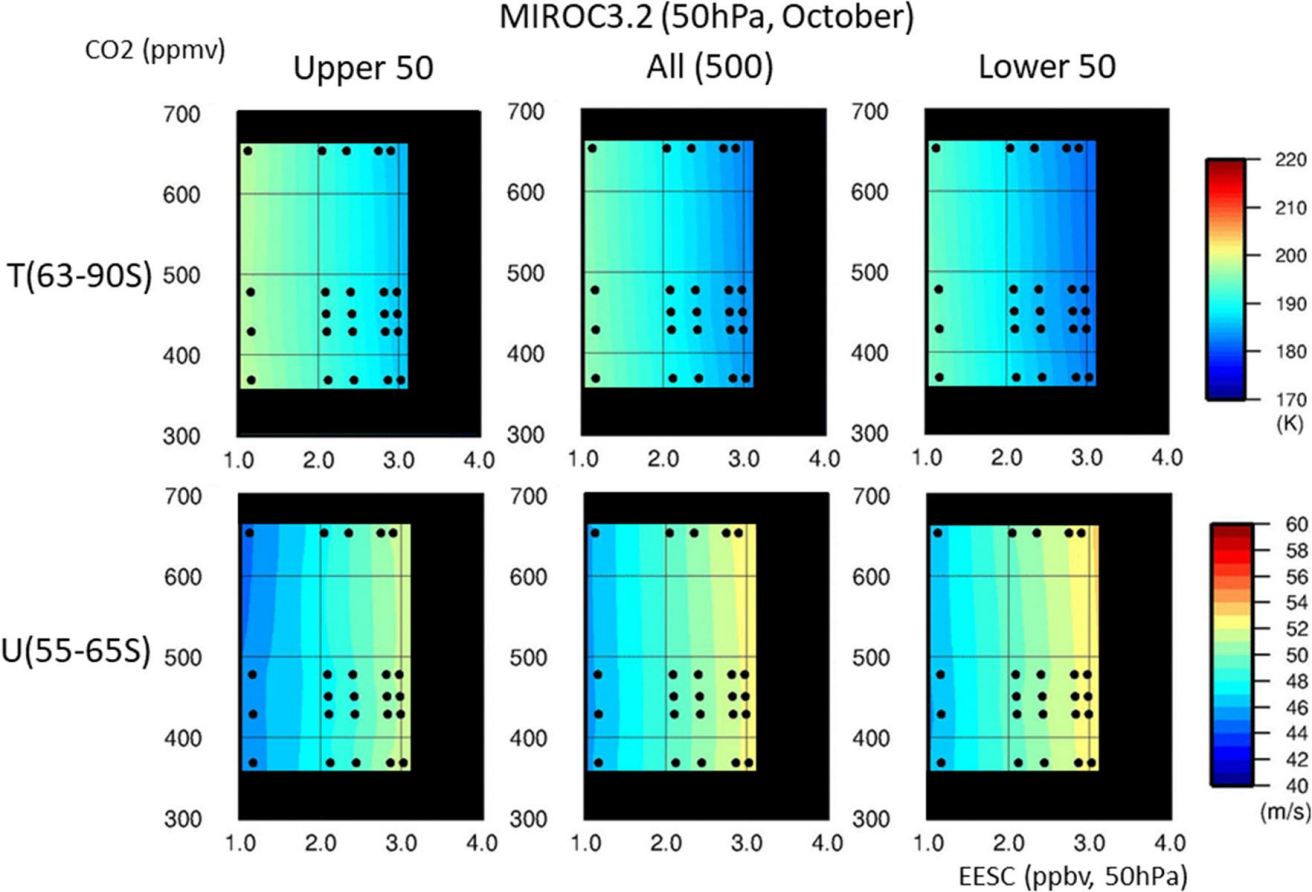
MIROC3.2 CCM results for the lower 50 ensemble members, full 500 members and upper 50 members regarding dependence of the polar cap temperature and polar night jet strength in the mid- and high latitudes of the SH (50 hPa, October) on ODS and GHG concentrations. The polar cap temperatures and polar night jet strength were calculated using the October mean values for daily temperature (63–90° S, 50 hPa) and daily zonal mean zonal wind speed (55–65° S, 50 hPa).

In the SH mid- and high latitudes, the column ozone minimum depended only on the ODS concentration for the means of the upper 50 members, the 500-member ensemble or the lower 50 members, as indicated by the almost vertical contours in the lower panels of Fig. 3. No obvious GHG dependence was observed for all three sets of members; similarly, the polar cap temperature and polar night jet strength in the lower stratosphere were ODS-dependent and almost GHG-independent for all three sets of members (Fig. 5). The difference in ODS and GHG dependence between the NH and SH was linked to the difference in stability between the polar vortices in each hemisphere, which results in a difference in wave activity. The Antarctic polar vortex is more stable and tighter than the Arctic polar vortex, and the transport of chemical constituents and heat is less effective in the Antarctic than in the Arctic. Thus, ozone in the Antarctic is more vulnerable to chemical destruction resulting from ODS increases.

### MIROC5 CCM

The results of the MIROC5 CCM were essentially similar to those of MIROC3.2 with regard to the ODS and GHG dependence of the spring column ozone minimum at mid- and high latitudes. However, there were several differences, including that MIROC5 consistently produced narrower distributions of the spring column ozone minimum in the NH than MIROC3.2, except for the four ODS-1960 pairings (see Supplementary Fig. S8). This is because spring total ozone in the Arctic was higher with MIROC5 than with MIROC3.2 (Supplementary Fig. S13). Furthermore, unlike with MIROC3.2, larger ODS amounts in the SH did not lead to narrower distributions with MIROC5 (Supplementary Fig. S9). Column ozone minima in both the NH and SH showed weaker ODS dependence with MIROC5 compared with MIROC3.2 (Supplementary Fig. S10). GHG dependence in the NH was weaker with MIROC5 than with MIROC3.2. In the SH, GHG dependence of total ozone was barely evident for both MIROC5 and MIROC3.2.

With regard to the ODS dependence of the Arctic polar cap temperature (63–90° N, 50 hPa) and zonal mean zonal wind (westerly, 60–70° N, 50 hPa) in March, results for the lower 50 ensemble members were similar to those of MIROC3.2. The higher the ODS concentration, the lower the polar cap temperature and the stronger the polar night jet. The upper 50 members did not show consistent ODS dependence between the two models (see Fig. 4 and Supplementary Fig. S11).

For the lower 50 ensemble members, the GHG dependence of polar cap temperature in the NH spring was weaker than that predicted by MIROC3.2, and that of polar night jet strength was almost the same as, or slightly weaker than, that predicted by MIROC3.2 (right panels in Fig. 4 and Supplementary Fig. S11), indicating that increased GHG led to a slightly weaker polar night jet and nearly unchanged polar cap temperature. This was also evident from the meridional distribution of the changes in zonal mean zonal wind (westerly) and zonal mean temperature associated with the GHG increase between 2000 and 2095 (middle panels of Supplementary Figs. S4 and S6). According to MIROC5, the GHG-associated differences among the lower 50 members with regard to Arctic temperature and wind at 50 hPa fell below the statistical significance level of 95%. The upper 50 members did not show consistent GHG dependence between the two models.

For the SH spring, MIROC5 indicated weaker ODS dependence for polar cap temperature and polar night jet strength than MIROC3.2 (Fig. 5 and Supplementary Fig. S12). GHG dependence of temperature and wind was not evident in the SH, except for polar night jet strength among the 500-member ensemble and the lower 50 (see Supplementary Fig. S12).

## Discussion

The results of the 500-member ensemble simulations using the MIROC3.2 and MIROC5 CCMs showed some common features regarding the ODS and GHG dependence of the spring column ozone minimum. The lower 50 ensemble members showed a clear ODS dependence. This implies that given the large interannual variations in ozone amounts in the NH mid- and high latitudes, severe Arctic ozone depletion could occur in a future year with a stable polar vortex if ODS concentrations remain high, even accounting for future increases in GHG concentration. In addition, both models found that an increase in GHG was associated with a small increase in the mean column ozone minimum for the upper 50 members, the lower 50 members and the 500-member ensemble in the NH.

According to the MIROC3.2 run using ODS and GHG values for the year 2000, the zonal mean column ozone values for the Arctic winter/spring had a minimum around March (Supplementary Fig. S13). This was due to the smaller magnitude of downward motion at high latitudes in winter/spring in MIROC3.2 than in MIROC5 (Supplementary Fig. S14). This implies that the Arctic polar vortex is more stable in the MIROC3.2 CCM than in the MIROC5 CCM, which leads to slightly different impacts on the ODS- and GHG-dependent variations in the spring column ozone minima. It is not easy to conclude whether the 500-member ensemble mean result from MIROC3.2 regarding the March minimum around 80–90° N is realistic in comparison with the 8-year average of Total Ozone Mapping Spectrometer (TOMS) observations, as TOMS observations were not available for polar winter. The MIROC3.2 results for ODS and GHG dependence may indicate possible conditions in the case of an Arctic polar vortex that is slightly more stable than the current polar vortex.

The fact that climate change has some effect on the Arctic severe ozone loss, in addition to the increased halogen loading in the atmosphere, has been discussed^18,19,60,61^. In terms of the effect of future global warming on an extreme ozone-destruction event in the Arctic, the lower 50 ensemble members from MIROC3.2 CCM and MIROC5 CCM indicate an increase in the column minimum ozone and temperature as GHG concentrations increase towards the end of the twenty-first century. This ozone and temperature dependence in the NH mid-and high latitude lower stratosphere on GHGs is different from the dependence reported by the local maxima of PSC formation potential (PFP^LM^) based on CMIP6 GCM output^19^, which shows a higher PFP^LM^ (lower temperature) towards the end of the twenty-first century in SSP5-8.5 and SSP3-7.0 scenarios, but similar to the result from the EMAC CCM on the point that cold winters do not increase towards the end of the twenty-first century^61^. The EMAC CCM result also shows that the temperature change is different between early winter and late winter/early spring. Thus, the dependences of ozone amount and temperature on GHG concentration in the NH mid- and high latitude lower stratosphere in the future are highly uncertain and model dependent, because the lower stratosphere is located near the boundary of radiative warming in the troposphere and radiative cooling in the stratosphere as GHGs increase, and because dynamical heating due to enhancement of the meridional circulation could occur in the mid- and high latitude lower stratosphere. The GHG response is a combined effect of these processes and, hence, complex. These processes include many model-dependent factors for simulation, such as the horizontal and vertical resolutions, the radiation parameters, and the gravity wave parameterization. The difference in GHG scenarios between our CCM (RCP-6.0) and other studies may cause some differences in the results. Furthermore, the temperature dependence in Fig. 4 is for a single pressure level (50 hPa) and month (March). The definition of PFP^LM^ includes temperatures at different levels in the lower stratosphere and other winter/early spring months.

If we consider only the lowest column ozone minimum among 500-member ensembles, some experiments show lower values in the GHG-2095 experiments than in the GHG-2000 experiments, which implies that a worse ozone-destruction event could occur in the future atmosphere with higher GHG concentrations (ODS-1995 experiments from MIROC3.2, and ODS-1985 and ODS-1995 experiments from MIROC5; see Fig. 1 and Supplementary Fig. S8). However, these are very rare cases (one or a few in 500 ensemble members) and ODS concentrations at the end of the twenty-first century will not be so high under future ODS regulations.

In conclusion, the average of the 50 ensemble members with the lowest column ozone in the mid- and high latitudes of the Northern Hemisphere from the two CCMs suggests that ODS concentrations should be reduced to avoid large ozone losses not only in the Antarctic but also in the mid- and high latitudes of the NH in the case of a stable Arctic polar vortex. It is unlikely on a once-in-a decade basis that GHG increase towards the end of the twenty-first century will cause a worse Arctic ozone-depletion event.

## Methods

The multi-ensemble simulations were initiated by setting the ODS and GHG surface concentrations to globally uniform, temporally constant values. A continuous 510-year calculation was then performed at these fixed ODS and GHG levels; for example, one of the runs consisted of a 510-year continuous calculation using ODS concentrations for 1995 under the WMO-A1 scenario and GHG concentrations for 2000 under the RCP 6.0 scenario. This run is presented in the bottom right corner of Table 1. The first 10 years were excluded from the analysis as preliminary outputs obtained before the ozone amount in the CCM reached a steady seasonal cycle at the specified ODS and GHG concentrations. The last 500 years were analysed as an ensemble of 500 members.

The MIROC3.2 and MIROC5 CCMs are based on versions 3.2 and 5 of the MIROC atmospheric general circulation model (AGCM), respectively, incorporating a common stratospheric chemistry module that was developed at NIES and the University of Tokyo. MIROC (Model for Interdisciplinary Research on Climate) was developed by the University of Tokyo, NIES and the Japan Agency for Marine-Earth Science and Technology^62–64^. The spatial resolution of the CCMs is a T42 spectral truncation (2.8° by 2.8°) in the horizontal direction, and the models have 34 vertical levels of hybrid sigma–pressure vertical coordinates from the surface to 0.01 hPa (approximately 80 km). The MIROC3.2 CCM performs simulations as recommended by the international Chemistry-Climate Model Initiative (CCMI) and its predecessor, Chemistry-Climate Model Validation 2 (CCMVal2) in order to project the future ozone layer and analyse the relationship between ozone changes and climate change. The MIROC5 CCM is a newly developed CCM intended to be coupled with an ocean model to study interactions between atmospheric composition and climate. However, in this study, as the MIROC3.2 CCM was not coupled with an ocean model, the MIROC5 CCM was also not coupled so as not to interfere with comparison of the results. Sea surface temperature (SST) and sea ice distributions were derived from external data, as described in the next section.

The MIROC3.2 and MIROC5 CCMs use a common stratospheric chemistry module with 42 photolysis reactions, 142 gas-phase chemical reactions and 13 heterogeneous reactions for multiple aerosol types^65,66^. Three types of polar stratospheric clouds (PSCs) were incorporated: a H_2_SO_4_-HNO_3_-H_2_O supercooled ternary solution (STS), nitric acid trihydrate (NAT) and ice (ICE). The reaction probabilities for STS were calculated using the scheme used for Arctic ozone loss simulation^67^, and those for NAT and ICE were obtained from JPL-2010^68^. These PSCs were assumed to have a single radius without size distribution. In this study, particle number density was assumed to be 10 particles/cm^3^ for STS, 1 particle/cm^3^ for NAT and 0.01 particles/cm^3^ for ICE. The value of the particle number density for STS was based on stratospheric aerosol observations^69^ and was derived as a number density parameter of the log-normal distribution (σ = 1.8). Those for NAT and ICE were based on the parameters used for the simulations^70,71^. Assuming a spherical configuration, PSC radius was calculated from the particle number density and condensation volume, with the latter calculated from the saturation vapour pressures of H_2_SO_4_, HNO_3_ and H_2_O. Then, the sedimentation velocities of the PSCs were calculated based on the radius as a function of pressure and temperature, using the Stokes terminal settling velocity with the Cunningham correction factor.

The photolysis rates of the chemical constituents were calculated online from the radiation flux in the CCMs, with 32 spectral bins for each CCM. The radiation fluxes and ozone concentrations in the models were consistent with each other, indicating an interaction between these parameters.

### Input data for CCM runs

The CCM calculations were based on yearly data on ODS and GHG concentrations at the surface. The ODSs included in the CCM were CFC-11, CFC-12, CFC-113, HCFC-22, CCl_4_, CH_3_Cl, CH_3_CCl_3_, Halon-1301, Halon-1211 and CH_3_Br; their surface concentrations were obtained from the WMO A-1 scenario. CHBr_3_ and CH_2_Br_2_ were also included for consistency with observations of the bromine budget in the stratosphere, giving approximately 21 pptv of Bry in the stratosphere around 2000. The GHGs included in the CCM were CO_2_, CH_4_ and N_2_O, and their surface concentrations were taken from the RCP 6.0 scenario. The CO_2_ concentration was assumed to be uniform not only at the surface but also throughout the model atmosphere, with the same mixing ratio as that at the surface. The surface concentrations of the other ODSs and GHGs were assumed to be horizontally uniform, as they were assumed to be transported to the troposphere, stratosphere, and mesosphere of the model, and degraded by photochemical reactions and reactions with free radicals. Assuming a horizontally uniform concentration of the ODSs and GHGs at the surface should not produce any serious errors in examining long-term, planetary-scale changes in ozone amount, as approximately 90% of column ozone exists in the stratosphere, and the actual ODS and GHG distributions at the surface are levelled out at the upper troposphere/lower stratosphere because of the large-scale transport and photochemical reactions in the troposphere.

The CCMs used monthly data for SST and sea ice from the Coupled Model Intercomparison Project Phase 5 (CMIP-5) simulations performed with the MIROC5 coupled atmosphere–ocean general circulation model. The monthly SST and sea ice data were averaged over 10 years. For example, the data average for 1995–2004 was used for the five GHG-2000 experiments (ODS-1960, 1980, 1985, 1990 and 1995), and the data average for 2025–2034 was used for the five GHG-2030 experiments. Therefore, the ENSO signals in the 10-year averaged data were removed. The solar flux data were based on the data for CCMI Phase 1 simulations, which were supplied by WCRP/SPARC SOLARIS-HEPPA^72^; however, in this study, the data average for 1960–2000 was used. Hence, the effects of interannual solar flux variations, such as the 11-year solar cycle, were removed. Furthermore, the MIROC3.2 and MIROC5 CCMs do not generate QBO internally. Thus, the simulations in this study did not include the effects of ENSO, solar flux and QBO.

### Output data from CCM runs

The CCMs output two-dimensional data (longitude/latitude) for column ozone and three-dimensional data for the ozone mixing ratio, temperature, zonal wind, meridional wind, air density and some other meteorological quantities and chemical constituents. In this study, we examined only the column ozone, temperature, zonal wind, total reactive chlorine concentration (Cly) and total reactive bromine concentration (Bry).

### Analysis of the spring column ozone minimum

The spring column ozone minimum in the NH mid- and high latitudes was determined by searching for the minimum value for each ensemble member in the region between 45° and 90° N from March to May. The amount of column ozone in the Arctic polar vortex is often low because of the catalytic destruction of ozone during these months. As the Arctic polar vortex shows large temporal and spatial variability, the region for analysis included not only the polar regions, but also the northern part of the mid-latitudes. In the SH, the spring minimum values for each ensemble member were determined in the region between 45° and 90° S from September to November, corresponding to the ozone hole months. A histogram of the spring ozone minimums of the 500-member ensembles was created for each experiment (Table 1). The horizontal axes of Figs. 3 and 4 represent average EESC at 50 hPa for the NH (45–90° N, March–May) and SH (45–90° S, September–November). The EESC was calculated by adding 65 times the reactive bromine concentration to the concentration of reactive chlorine.

### Analysis of the area with column ozone of less than 220 DU

Because the column ozone bias of the MIROC3.2-CCM and MIROC5-CCM, based on TOMS observations, is not large globally (see Supplementary Fig. S13), we used a threshold of 220 DU, the ozone value indicating an ozone hole, to determine the ozone loss area for both models. The grids that indicated column ozone amounts of less than 220 DU were taken from daily column ozone data for the area between 45° and 90° N from March to May and the area between 45° and 90° S from September to November, and the daily values of the areas of the low-ozone grids were integrated with respect to the day for the three months of spring. Then, the ensemble distribution of the values was calculated, with units of 10^8^ km^2^·day (see Supplementary Figs. S2 and S3).

### Polar cap temperature and zonal mean zonal wind at 50 hPa

The Arctic polar cap temperature and zonal mean zonal wind at 50 hPa (Fig. 4) were calculated by averaging the daily temperature data in the polar cap region (63–90° N; model grids of 9 points on latitude by 128 points on longitude) and the daily zonal wind data in the zonal wind maximum region (60–70° N; grids of 5 points on latitude by 128 points on longitude), respectively, during March. Similarly, the Antarctic polar cap temperature and zonal mean zonal wind at 50 hPa (Fig. 5) were calculated by averaging the daily temperature data in the polar cap region (63°–90° S; model grids of 9 points on latitude by 128 points on longitude) and the daily zonal wind data in the zonal wind maximum region (55–65° S; grids of 5 points on latitude by 128 points on longitude), respectively, during October.

## Supplementary Information

Supplementary Information.

## Data Availability

The model output used in this study are available from https://doi.org/10.17595/20221109.001.
