# Supplementary material for: Dependence of column ozone on future ODSs and GHGs in the variability of 500-ensemble members
Source: Sci Rep. 2023 Jan 6;13:320. doi: 10.1038/s41598-023-27635-y (PMC9822909; doi:10.1038/s41598-023-27635-y)
Supplement: Supplementary file 1 — Supplementary Information. [file 41598_2023_27635_MOESM1_ESM.docx]

Supplementary Information:

Supplementary Results: Time-integrated area with spring column ozone less than 220 DU in the regions between 45° and 90°N/S.

Because the spring column ozone minimum is a localized and instantaneous parameter, it may not comprehensively represent spring ozone depletion over an entire region and time period. Thus, we calculated the area with column ozone values less than 220 DU in the region between 45° and 90°N (Supplementary Fig. 1) and the time-integrated area of ozone loss during the period from March to May (Supplementary Fig. 2) in order to examine the consistency of ODS and GHG dependence between the column ozone minimum and the time-integrated ozone loss area. The time-integrated area between 45° and 90°S from September to November is shown in Supplementary Fig. 3. The frequency distribution of the values for the day-integrated ozone loss area differed notably between NH and SH. In the NH, the distribution showed more ensemble members with smaller area values and fewer ensemble members with larger values, whereas in the SH, the distribution peaked with medium values. The difference is likely due to the difference in ozone destruction potential between the Arctic and Antarctic polar vortices. In the NH, because of the high variability of the Arctic polar vortex in winter/spring, ozone destruction is limited temporally and spatially and much lower than that in the SH. The Arctic polar vortex showed high variability among the ensemble members. For some members, the Arctic polar vortex was stable in winter/spring, and the temperature inside the vortex is low, leading to chemical ozone destruction inside the vortex; for other members, the Arctic polar vortex was unstable and the temperature was high, preventing chemical ozone destruction and resulting in the effective transport of ozone from mid-latitudes to the Arctic. On the other hand, a large spring ozone loss occurred in the Antarctic polar vortex for almost all ensemble members, with low variability among members. These differences resulted in a large difference in the frequency distribution of ozone loss area values between the NH and SH.

Supplementary Results: ODS and GHG dependence of polar cap temperature and zonal mean zonal wind in meridional sections of the NH and SH springs

Because the data for the ODS and GHG dependence of polar cap temperature and zonal mean zonal wind shown in Figs. 4 and 5 are derived only from selected grid points of the model in the high-latitude lower stratosphere (63–70°N/S and 50 hPa for the polar cap temperature, 60–70°N and 50 hPa for the NH zonal mean zonal wind, 55–65°S and 50 hPa for the SH zonal mean zonal wind), it is useful to examine ODS and GHG dependence over the entire meridional section, although only a limited number of experimental results are shown in the figures.

The left panels of Supplementary Fig. 4 show the meridional distribution, by contours, of the zonal mean temperature in March for the 50 ensemble members with the lowest total ozone values from the ODS-1995&GHG-2000 pairing, as predicted by the MIROC3.2 CCM, in comparison with other experiments (ODS-1960&GHG-2000 for the upper panel, ODS-1995&GHG-2095 for the middle panel and ODS-1960&GHG-2095 for the lower panel). Differences from the ODS-1995&GHG-2000 pairing are indicated by colour. These four pairings correspond to the black dots at the four corners in Fig. 4 (bottom right, ODS-1995&GHG-2000; bottom left, ODS-1960&GHG-2000; top right, ODS-1995&GHG-2095; top left, ODS-1960&GHG-2095). Colour differences in the upper panel show the effects of decreasing ODS, those in the middle panel show the effects of increasing GHG, and those in the lower panel show the effects of both. The results in Supplementary Fig. 4 and Supplementary Fig. 5 regarding ODS and GHG dependence of the polar cap temperature around 50 hPa are consistent with those in Figs. 4 and 5; in the NH spring, the future ODS decrease increased the polar cap temperature (top panel of Supplementary Fig. 4), and the future GHG increase also increased it (middle panel). In the SH spring, the future ODS decrease increased the polar cap temperature (top panel of Supplementary Fig. 5), but the future GHG increase slightly decreased it (middle panel). For the NH spring, GHG dependence of the polar cap temperature at 50 hPa had the opposite sign from that in the middle and upper stratosphere, whereas for the SH spring, it had the same sign.

The left panels of Supplementary Fig. 6 show the meridional distribution, indicated by contours, of the zonal mean zonal wind in March for the lower 50 ensemble members from the MIROC3.2 CCM using the ODS-1995&GHG-2000 pairing, as well as comparing this pairing with others (ODS-1960&GHG-2000 for the upper panel, ODS-1995&GHG-2095 for the middle panel and ODS-1960&GHG-2095 for the lower panel), with differences indicated by colour. The figure indicates that in the NH spring, the modelled future ODS decrease decelerated the polar night jet (top panel), and the future GHG increase decelerated it and accelerated the upper part of the subtropical jet (middle panel), resulting in a combined effect of decelerating the polar night jet and accelerating the subtropical jet (bottom panel). In the SH spring (Supplementary Fig. 7), the same effects due to ODS and GHG were evident, although the deceleration of the polar night jet by the GHG increase was very weak. These meridional variations among the four pairings with regard to the zonal mean zonal wind in the stratosphere are consistent with the results regarding ODS and GHG dependence of the wind in the high-latitude lower stratosphere shown for the lower 50 group in Figs. 4 and 5 (bottom right panels); on the panel for the NH spring, the zonal mean zonal winds decelerated in the leftward (ODS decrease) and upward (GHG increase) directions (Fig. 4), but decelerated only in the leftward direction for the SH spring (Fig. 5).

Supplementary Results: Results from MIROC5 CCM

The spring column ozone minimum distributions of the 500-member ensembles from the MIROC5 CCM are shown in Supplementary Figs. 8 and 9, which correspond to Figs. 1 and 2 for the MIROC3.2 CCM, respectively.

Supplementary Fig. 10 shows the MIROC5 CCM results regarding the ODS and GHG dependence of the ensemble-mean spring column ozone minimum values at mid- and high latitudes of both hemispheres, corresponding to Fig. 3 for MIROC3.2. Supplementary Fig. 11 shows the MIROC5 CCM results regarding the ODS and GHG dependence of the Arctic polar cap temperature (63–90°N, 50 hPa) and the zonal mean zonal wind (60–70°N, 50 hPa; polar night jet) for March, corresponding to Fig. 4 for MIROC3.2. Supplementary Fig. 12 shows the MIROC5 CCM results regarding the ODS and GHG dependence of the Antarctic polar cap temperature (63–90°S, 50 hPa) and zonal mean zonal wind (55°–65°S, 50 hPa; polar night jet) for October, corresponding to Fig. 5 for MIROC3.2.

The MIROC5 results regarding the ODS and GHG dependence of the polar cap temperature and zonal mean zonal wind throughout full meridional sections of the NH and SH spring are shown in the right columns of Supplementary Figs. 4–7. In addition to the difference between the two models in the results of the ODS-1995&GHG-2000 pairing, another obvious difference is that MIROC5 returned a much smaller value than MIROC3.2 for GHG-influenced zonal mean zonal wind deceleration in the NH in March (middle panels in Supplementary Fig. 6), and warming of the polar cap temperature in the lower stratosphere was not as evident as with MIROC3.2 (middle panels in Supplementary Fig. 4).

**Supplementary Discussion: Comparisons of zonal mean column ozone and the vertical component of the residual mean circulation between MIROC3.2 and MIROC5 CCMs.**

ODS and GHG dependence of the spring column minimum ozone is affected by polar vortex strength, which is related to the strength of the residual mean circulation in the winter and spring. Strong downward motion in the polar stratosphere corresponds to a weak and unstable polar vortex, whereas weak downward motion corresponds to a strong and stable polar vortex. Thus, the difference between the models with regard to the residual mean circulation in the polar winter/spring produced different results for the column ozone amount and for ODS and GHG dependence. Supplementary Fig. 13 shows time–latitude cross sections of zonal mean column ozone distribution from TOMS observations, MIROC3.2 CCM and MIROC5 CCM around the year 2000. The TOMS column ozone value is the 8-year average for 1997–2004, whereas the MIROC values are 500-member ensemble means based on the ODS-2000&GHG-2000 run. Supplementary Fig. 14 shows the distributions of the vertical component of the residual mean circulation at 50 hPa as predicted by the two CCMs, as well as the difference between them (MIROC5 – MIROC3.2). The difference figure illustrates that MIROC5 modelled stronger downward motion in the Arctic region from December to March than MIROC3.2, resulting in a higher value for Arctic spring ozone.

**Supplementary Figures:**

**
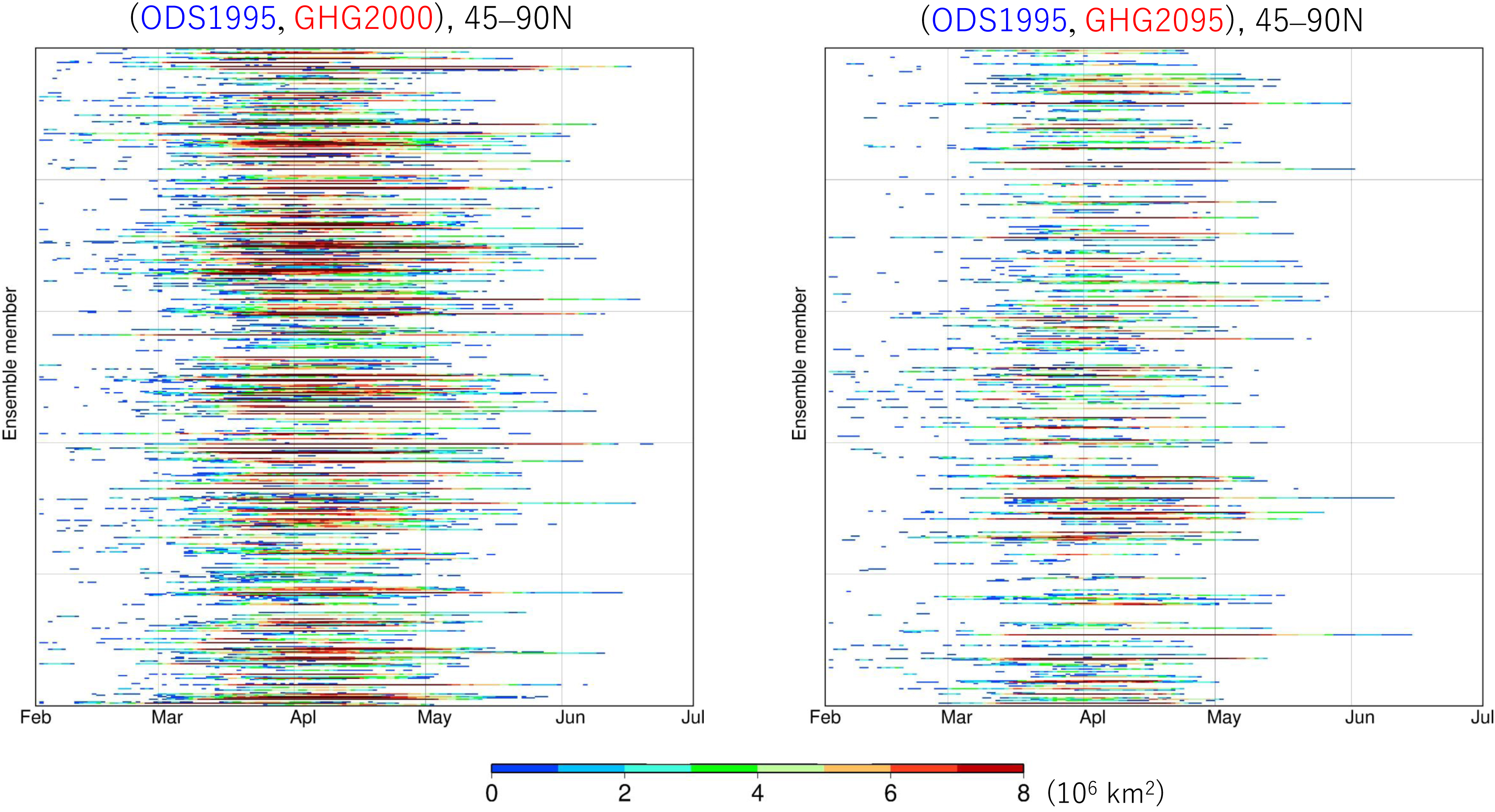
**

**Supplementary Fig. 1. MIROC3.2 CCM results for 500-member ensembles regarding area with column ozone less than 220 DU in the NH (45–90°N) as a function of day for the ODS-1995&GHG-2000 (left panel) and ODS-1995&GHG-2095 (right panel) runs.** Each ensemble member is shown on the vertical axis, with ensemble member 1 at the bottom and ensemble member 500 at the top. For each ensemble member, the area with column ozone less than 220 DU is indicated by colour, with units of 10^6^ km^2^, as a function of day from 1 February to 30 June. White indicates an area with column ozone more than 220 DU.

**MIROC3.2, 45-90N**


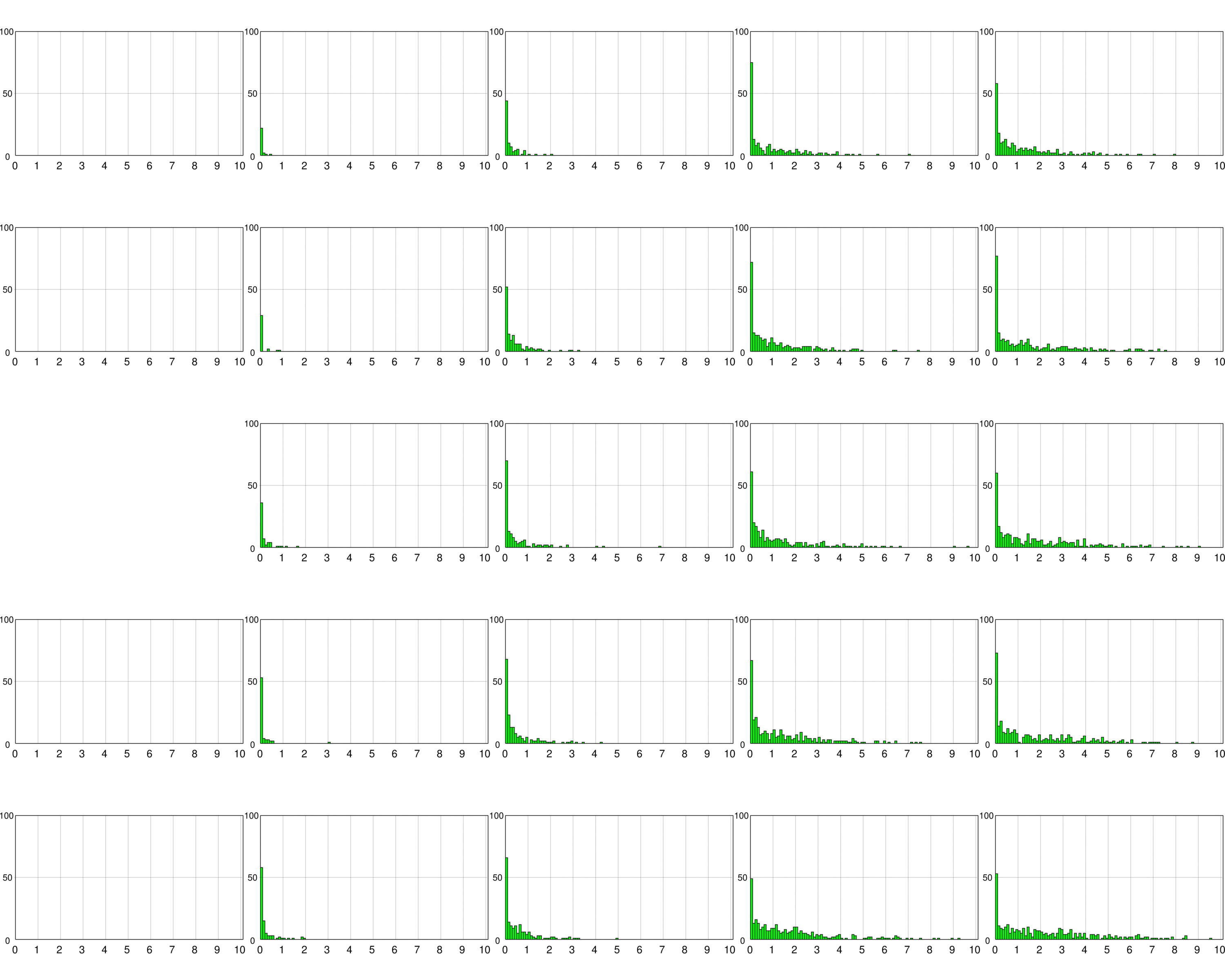


ODS-1960

ODS-1980

ODS-1985

ODS-1990

ODS-1995

GHG-

2095

GHG-

2050

GHG-

2040

GHG-

2030

GHG-

2000

**Supplementary Fig. 2. Histogram of day-integrated areas with column ozone less than 220 DU during the NH spring (45–90°N, March–May) for all 24 MIROC3.2 CCM runs with 500-member ensembles.** Results for all runs are shown in panels arranged according to the different ODS and GHG concentrations. The panels are arranged in the same order as in Table 1: the top row is for the GHG-2095 experiments, the bottom row the GHG-2000 experiments, the left column the ODS-1960 experiments and the right column the ODS-1995 experiments. The units for the horizontal axis are 10^8^ km^2^·day. The bin size on the horizontal axis is 0.2 × 10^8^ km^2^ ·day, with the bin of the smallest value at 0 and that of the largest value at 10 × 10^8^ km^2^·day. Bin count (between 0 and 100) is indicated on the vertical axis.

**MIROC3.2, 45-90S**


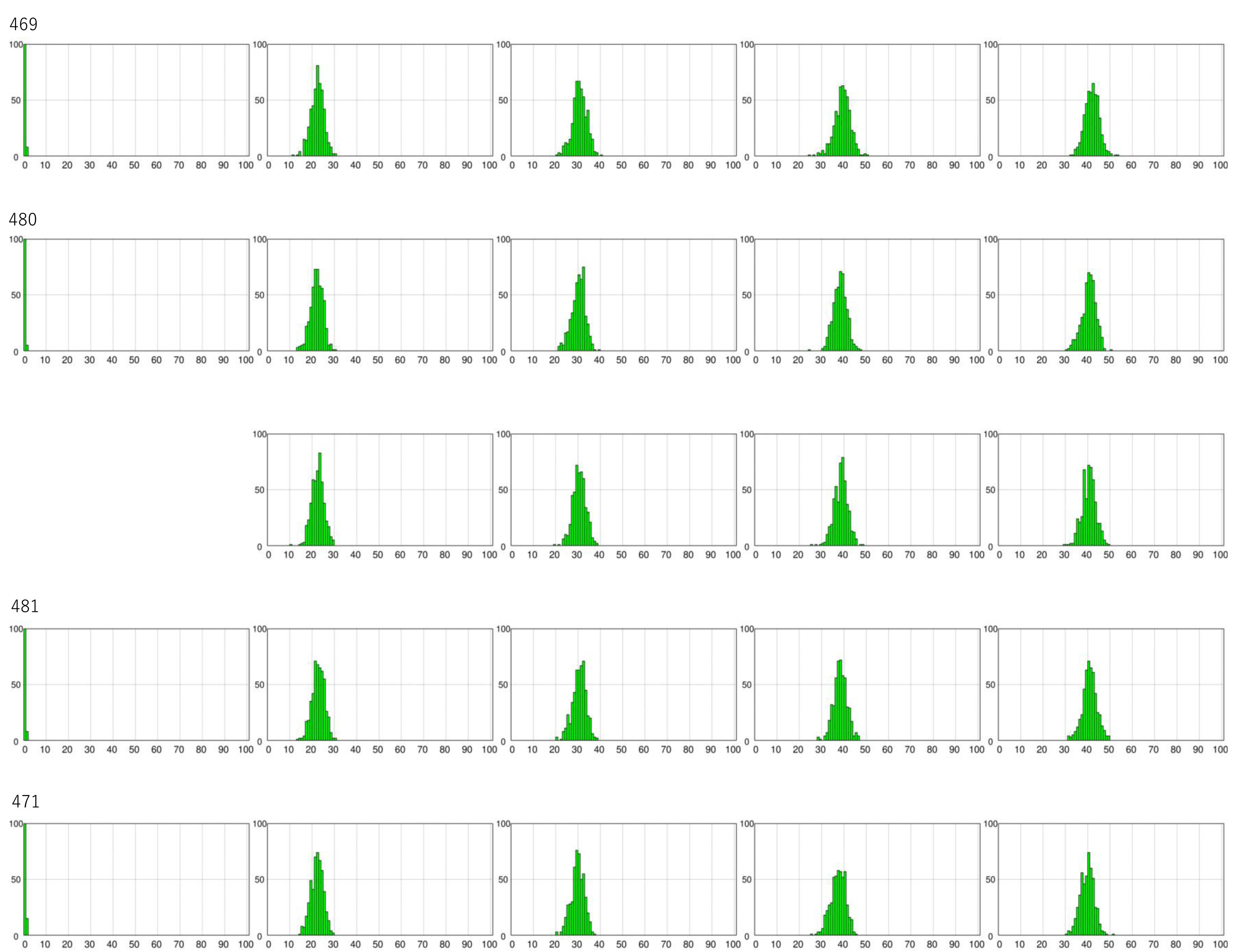


ODS-1960

ODS-1980

ODS-1985

ODS-1990

ODS-1995

GHG-

2095

GHG~~-~~

2050

GHG~~-~~

2040

GHG-

2030

GHG-

2000

**Supplementary Fig. 3. Histogram of day-integrated areas with column ozone less than 220 DU during the SH spring (45–90°S, September–November) for all 24 MIROC3.2 CCM runs with 500-member ensembles.** Results for all runs are shown in panels arranged according to the different ODS and GHG concentrations. The panels are arranged in the same order as in Table 1: the top row is for the GHG-2095 experiments, the bottom row the GHG-2000 experiments, the left column the ODS-1960 experiments and the right column the ODS-1995 experiments. The units for the horizontal axis are 10^8^ km^2^·day. The bin size on the horizontal axis is 2 × 10^8^ km^2^·day, with the bin of the smallest value at 0 and that of the largest value at 100 × 10^8^ km^2^·day. Bin count (between 0 and 100) is indicated on the vertical axis. Note that bin counts of the 0–2 × 10^8^ km^2^·day bins in the left column panels for the ODS-1960 experiments exceed 100; the values are indicated above the panels.


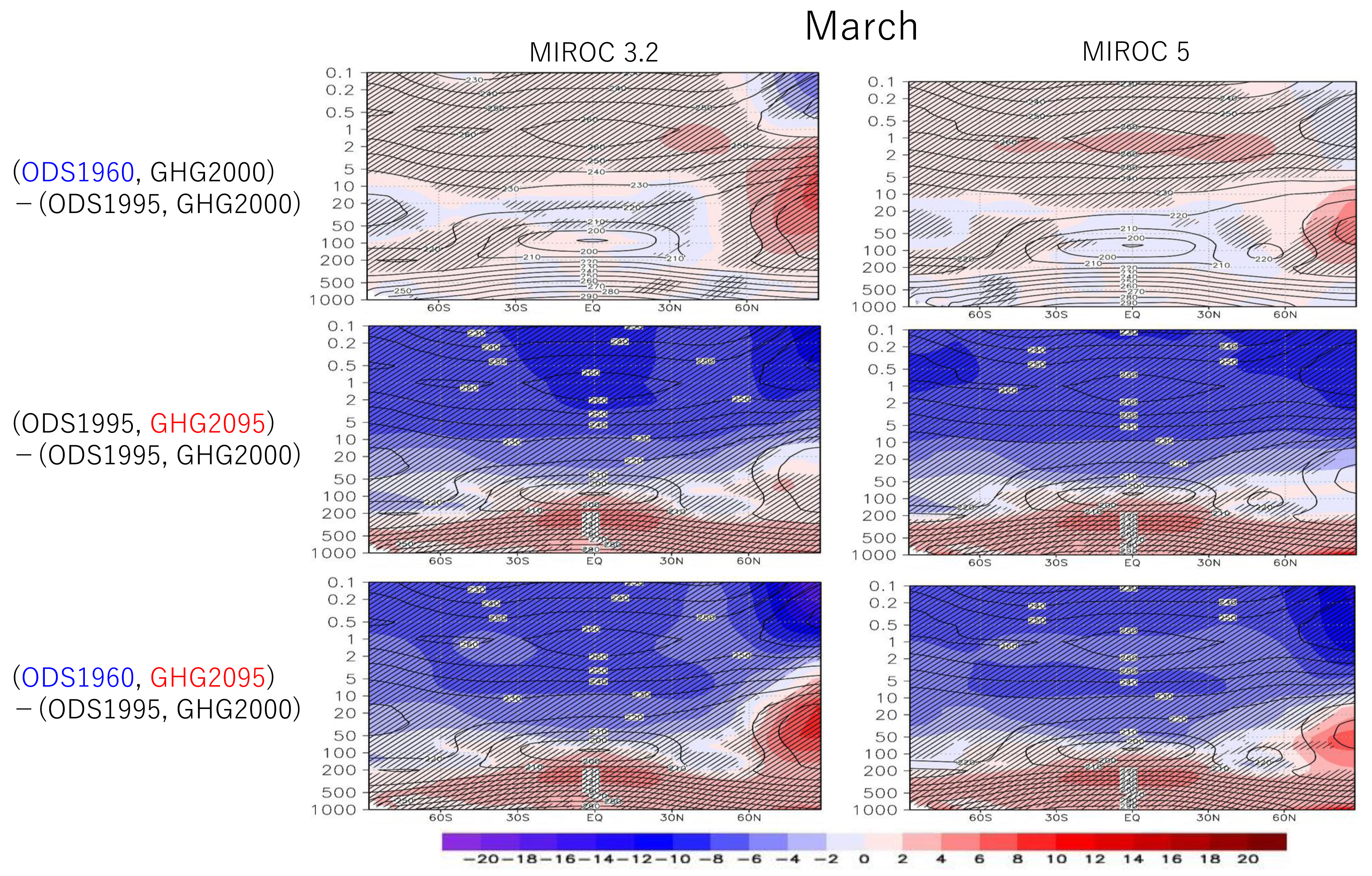


**Supplementary Fig. 4. Meridional distribution of March zonal mean temperature anomalies from the lower 50 members of the ODS-1995&GHG-2000 ensemble for MIROC3.2-CCM (left) and MIROC5-CCM (right).** Colours indicate differences in zonal mean temperature between ODS/GHG pairings: ODS-1960&GHG-2000 subtracted from ODS-1995&GHG-2000 (top, the ODS-decrease effect), ODS-1995&GHG-2095 subtracted from ODS-1995&GHG-2000 (middle, the GHG-increase effect), and ODS-1960&GHG-2095 subtracted from ODS-1995&GHG-2000 (bottom, combined ODS-decrease and GHG-increase effects). Red indicates a positive anomaly (warming) and blue indicates a negative anomaly (cooling). The contours indicate the March zonal mean temperature for the ODS-1995&GHG-2000 run. Shading indicates the regions where statistical significance level by *t*-test is 95% or more. GMT was used for making figures.


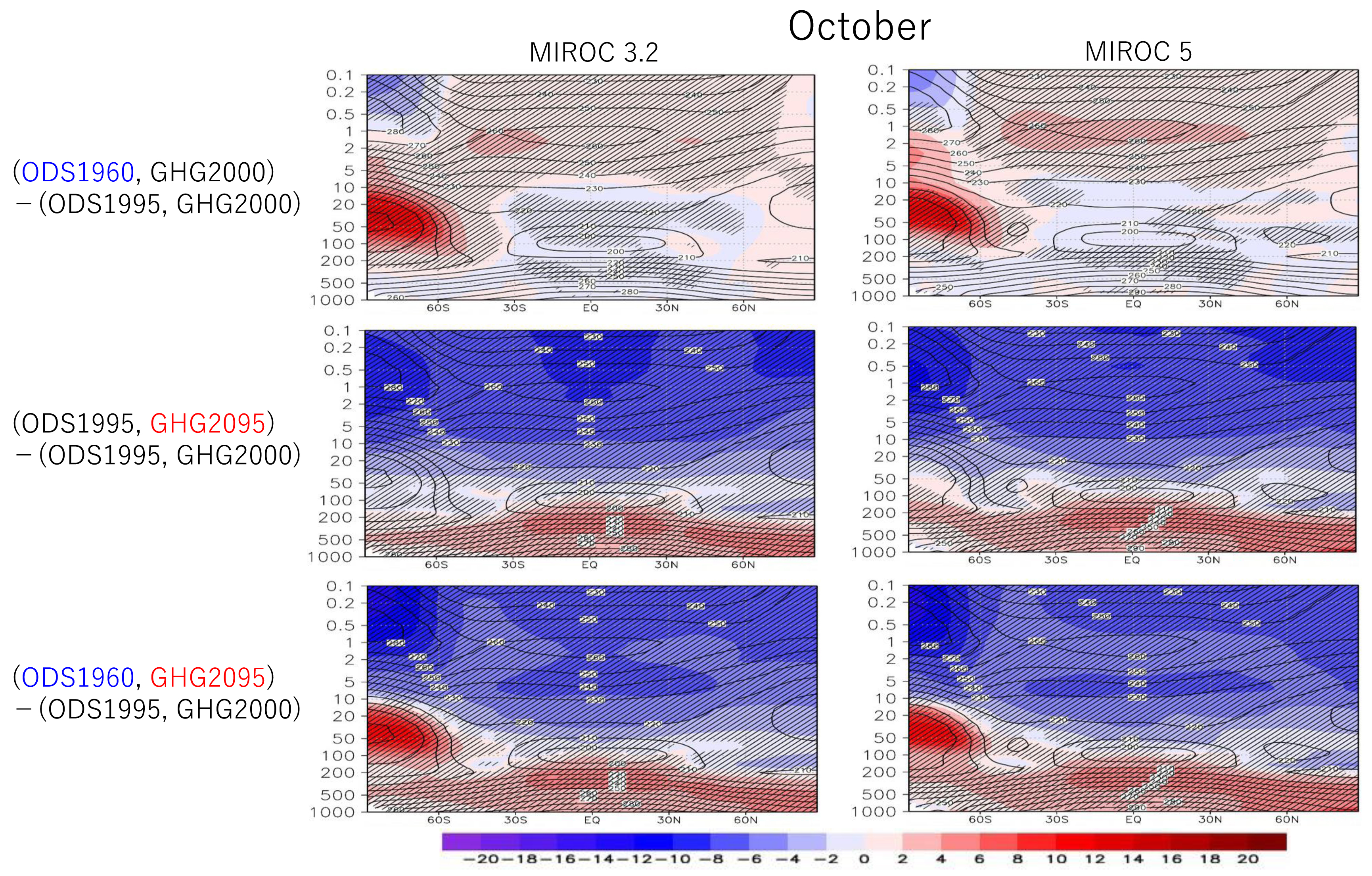


**Supplementary Fig. 5. Meridional distribution of October zonal mean temperature anomalies from the lower 50 members of the ODS-1995&GHG-2000 ensemble for MIROC3.2-CCM (left) and MIROC5-CCM (right).** Colours indicate differences in zonal mean temperature between ODS/GHG pairings: ODS-1960&GHG-2000 subtracted from ODS-1995&GHG-2000 (top, the ODS-decrease effect), ODS-1995&GHG-2095 subtracted from ODS-1995&GHG-2000 (middle, the GHG-increase effect), and ODS-1960&GHG-2095 subtracted from ODS-1995&GHG-2000 (bottom, combined ODS-decrease and GHG-increase effects). Red indicates a positive anomaly (warming) and blue indicates a negative anomaly (cooling). The contours indicate the October zonal mean temperature for the ODS-1995&GHG-2000 run. Shading indicates the regions where statistical significance level by *t*-test is 95% or more. GMT was used for making figures.


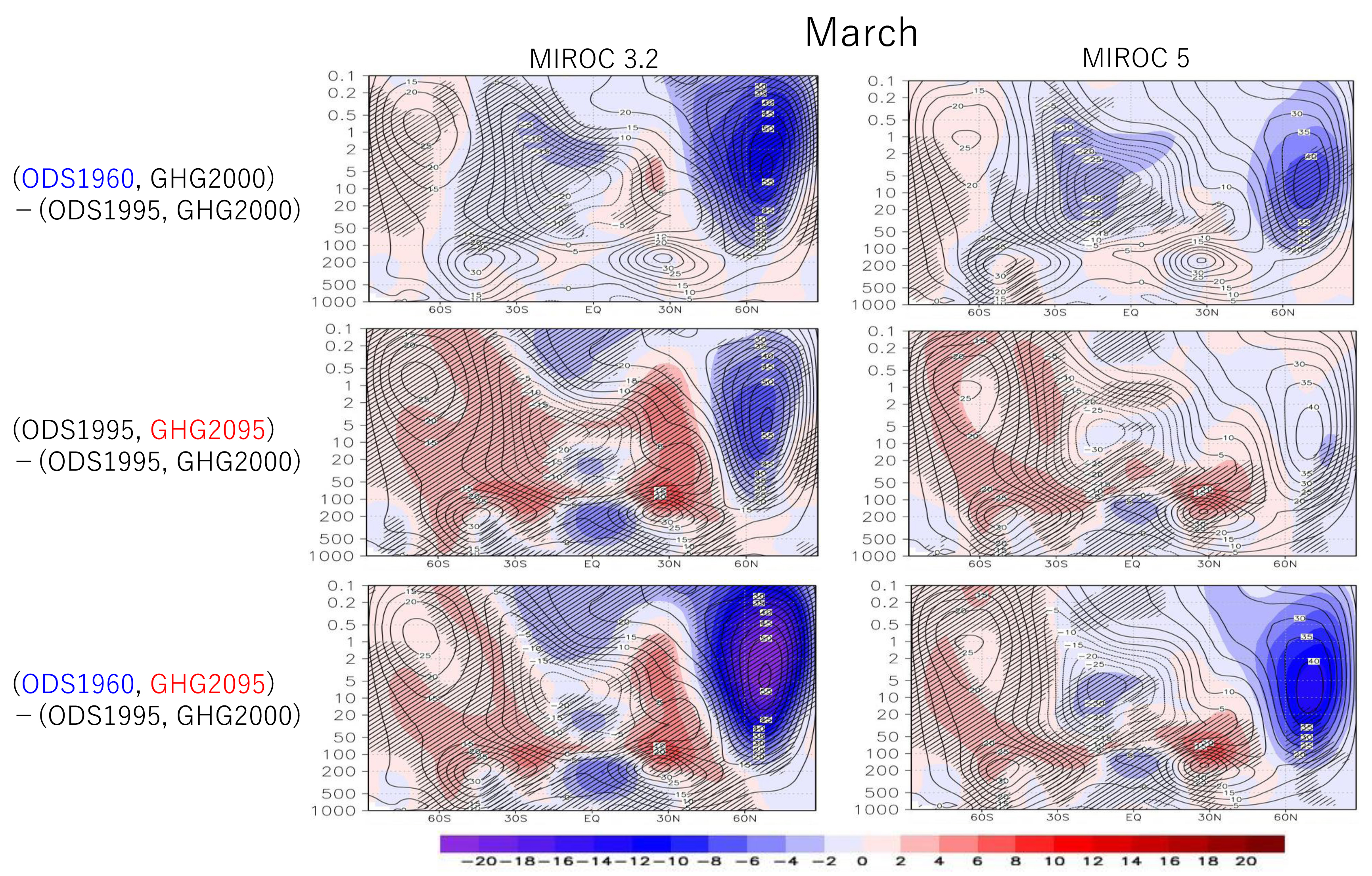


**Supplementary Fig. 6. Meridional distribution of March zonal mean zonal wind anomalies from the lower 50 members of the ODS-1995&GHG-2000 ensemble for MIROC3.2-CCM (left) and MIROC5-CCM (right).** Colours indicate differences in zonal mean zonal wind between ODS/GHG pairings: ODS-1960&GHG-2000 subtracted from ODS-1995&GHG-2000 (top, the ODS-decrease effect), ODS-1995&GHG-2095 subtracted from ODS-1995&GHG-2000 (middle, the GHG-increase effect), and ODS-1960&GHG-2095 subtracted from ODS-1995&GHG-2000 (botom, combined ODS-decrease and GHG-increase effects). Red indicates a westerly anomaly and blue indicates an easterly anomaly. The contours indicate the March zonal mean zonal wind for the ODS-1995&GHG-2000 run. Shading indicates the regions where statistical significance level by *t*-test is 95% or more. GMT was used for making figures.


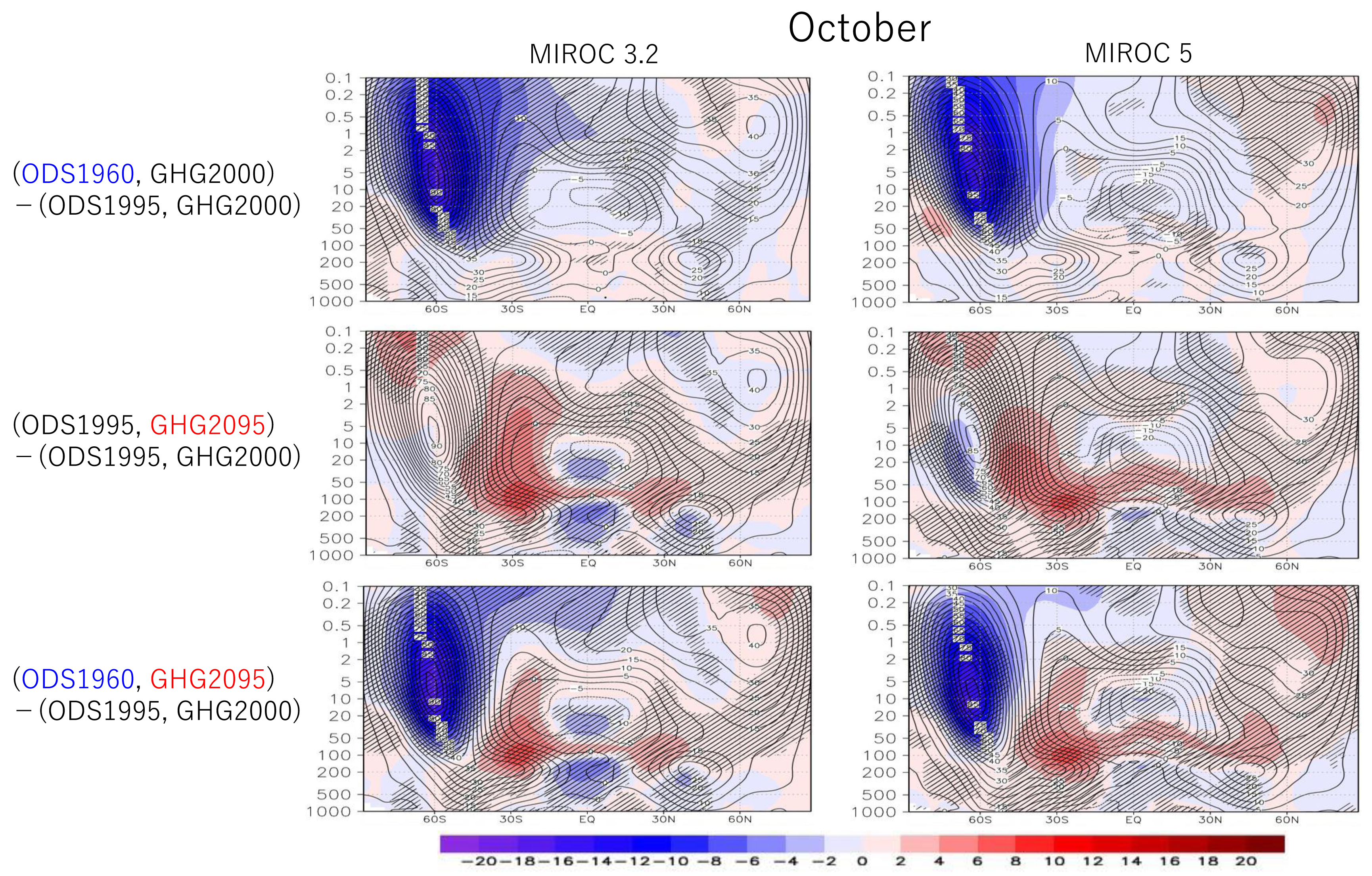


**Supplementary Fig. 7. Meridional distribution of October zonal mean zonal wind anomalies from the lower 50 members of the ODS-1995&GHG-2000 ensemble for MIROC3.2-CCM (left) and MIROC5-CCM (right).** Colours indicate differences in zonal mean zonal wind between ODS/GHG pairings: ODS-1960&GHG-2000 subtracted from ODS-1995&GHG-2000) (top, the ODS-decrease effect), ODS-1995&GHG-2095 subtracted from ODS-1995&GHG-2000 (middle, the GHG-increase effect), and ODS-1960&GHG-2095 subtracted from ODS-1995&GHG-2000 (bottom, combined ODS-decrease and GHG-increase effects). Red indicates a westerly anomaly and blue indicates an easterly anomaly. The contours indicate the October zonal mean zonal wind for the ODS-1995&GHG-2000 run. Shading indicates the regions where statistical significance level by *t*-test is 95% or more. GMT was used for making figures.

**MIROC5, 45-90N**


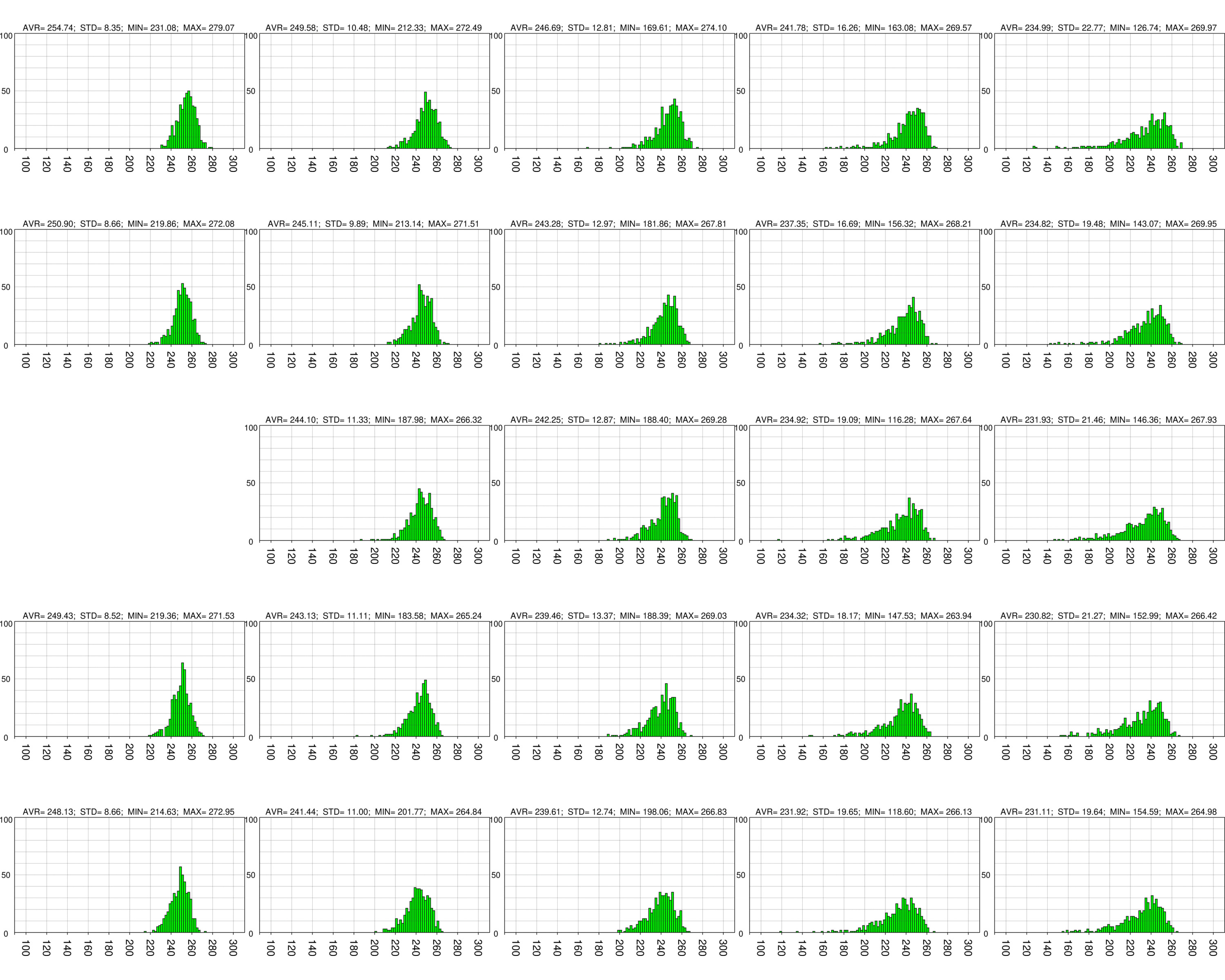


ODS-1960

ODS-1980

ODS-1985

ODS-1990

ODS-1995

GHG-

2095

GHG~~-~~

2050

GHG-

2040

GHG-

2030

GHG-

2000

**Supplementary Fig. 8. Histograms of the spring column ozone minimum values between 45° and 90°N from March to May for the 24 MIROC5 CCM runs with 500-member ensembles.** Results are shown by panels for all runs using different ODS and GHG concentrations. The panels are arranged in the same order as in Table 1: the top row shows the GHG-2095 experiments, the bottom row the GHG-2000 experiments, the left column the ODS-1960 experiments, and the right column the ODS-1995 experiments. The spring column ozone minimum values are depicted on the horizontal axis at intervals of 20 DU. The size of each bin for the column ozone minima on the horizontal axis is 2 DU, with the bin of the smallest value at 90–92 DU and that of the largest value at 308–310 DU. The bin count (between 0 and 100) is indicated on the vertical axis. Column ozone values (average with standard deviation, maximum and minimum) for the 500-member ensembles of each experiment are represented in the upper parts of the panels.

**MIROC5, 45-90S**


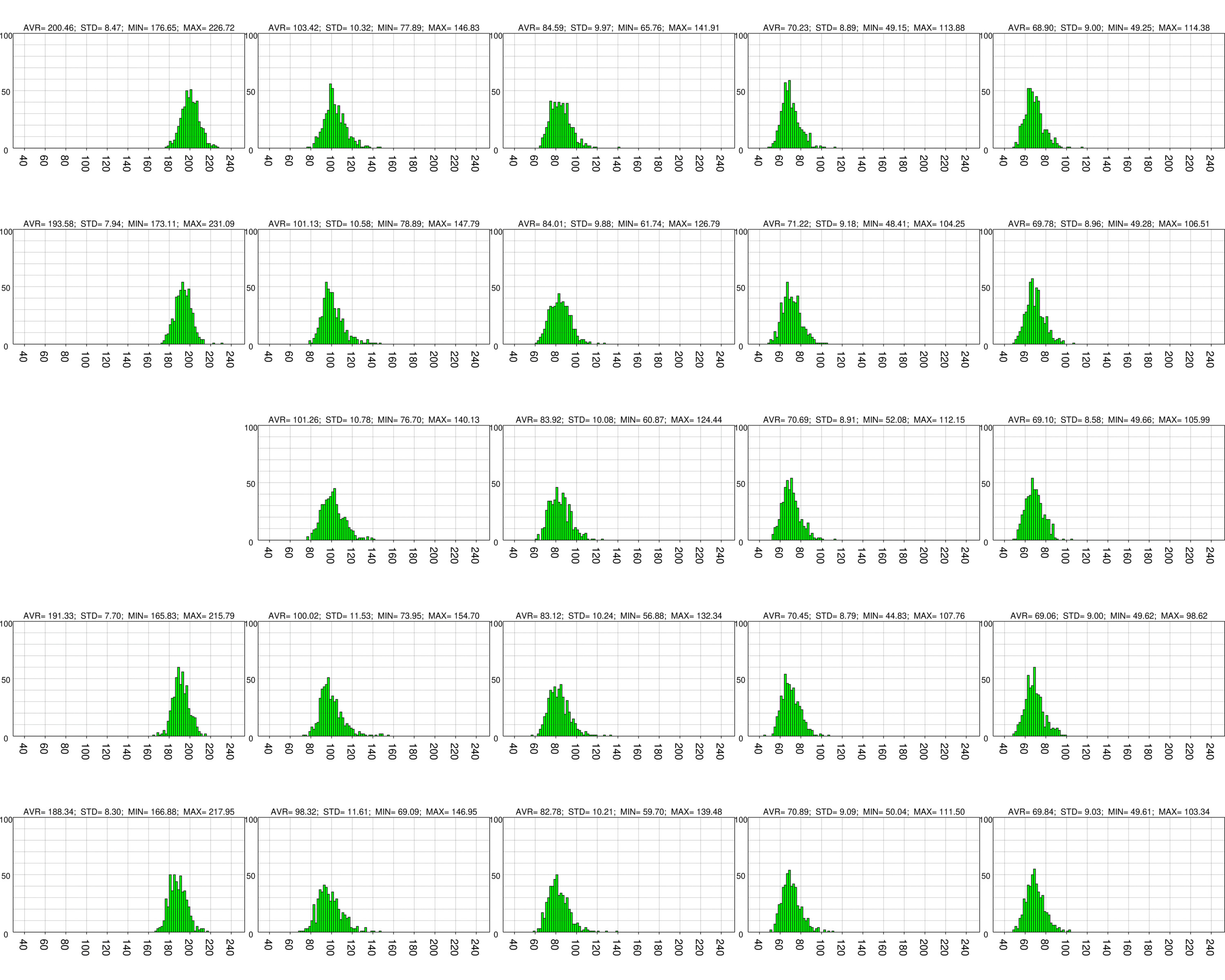


ODS-1960

ODS-1980

ODS-1985

ODS-1990

ODS-1995

GHG-

2095

GHG-

2050

GHG-

2040

GHG-

2030

GHG-

2000

**Supplementary Fig. 9. Histogram of the spring column ozone minimum values between 45° and 90°S from September to November for the 24 MIROC5 CCM runs with 500-member ensembles.** Results are shown by panels for all runs using different ODS and GHG concentrations. The panels are arranged in the same order as in Table 1: the top row shows the GHG-2095 experiments, the bottom row the GHG-2000 experiments, the left column the ODS-1960 experiments, and the right column the ODS-1995 experiments. The spring column ozone minimum values are depicted on the horizontal axis at intervals of 20 DU. The bin size for the column ozone minima on the horizontal axis is 2 DU, with the bin of the smallest value at 30–32 DU and the largest at 248–250 DU. The bin count (between 0 and 100) is indicated on the vertical axis. Column ozone values (average with standard deviation, maximum and minimum) for the 500-member ensembles of each experiment are represented in the upper parts of the panels.


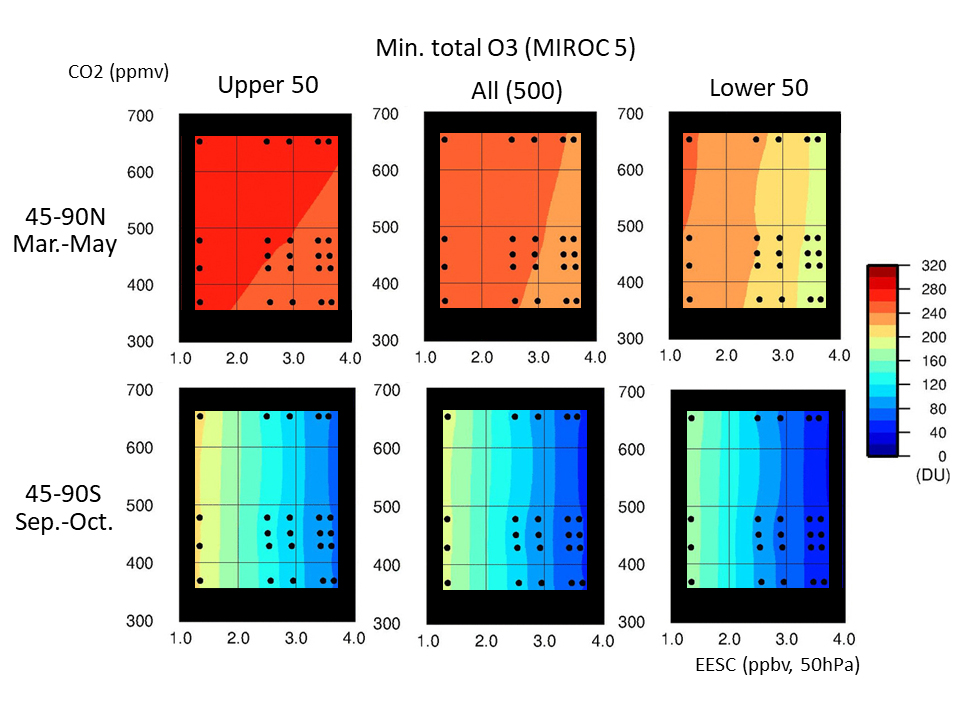


**Supplementary Fig. 10. MIROC5 CCM results for the lower 50 ensemble members, full 500 members and upper 50 members regarding dependence of spring column ozone minimum values in the mid- and high latitudes on ODS and GHG concentrations.** The upper panels show results for the area between 45° and 90°N from March to May (NH spring), and the lower panels show results for the area between 45° and 90°S from September to November (SH spring). Column ozone minimum values are averaged over the 50 ensemble members with the largest column ozone minima (upper 50, left), the full ensemble (500 members, middle) and the 50 members with the smallest column ozone minima (lower 50, right). The horizontal axis shows the mean spring EESC concentrations of the ODSs in ppbv for the NH (45–90°N, 50 hPa, March–May) and SH (45–90°S, 50 hPa, September–November). The vertical axis denotes the CO_2_ concentration in ppmv. Black circles denote the EESC and CO_2_ concentrations used in the 24 CCM runs. Colour levels represent column ozone minimum values in DU. The column ozone minimum values obtained from the 24 runs are interpolated and extrapolated in the (EESC, CO_2_) space. Most of the extrapolated regions are masked with black.


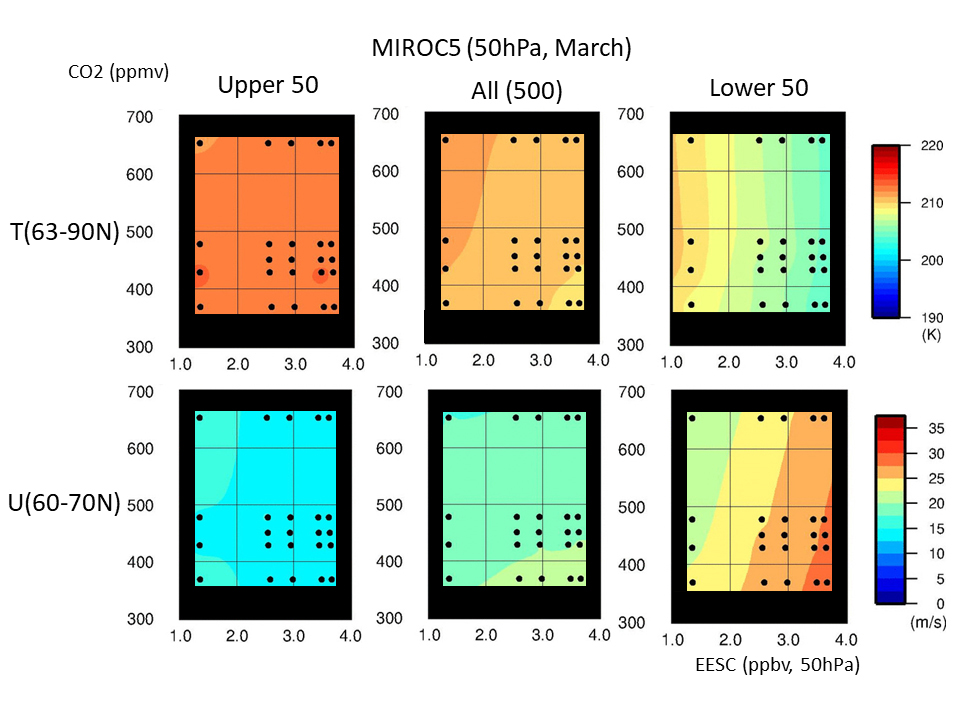


**Supplementary Fig. 11. MIROC5 CCM results for the lower 50 ensemble members, full 500 members and upper 50 members regarding dependence of the polar cap temperature and polar night jet strength in the mid- and high latitudes of the NH (50 hPa, March) on ODS and GHG concentrations.** The polar cap temperatures and polar night jet strength were calculated using the March mean values for daily temperature (63–90°N, 50 hPa) and daily zonal mean zonal wind speed (60–70°N, 50 hPa). Colour levels represent the temperature (in K) and zonal mean zonal wind speed (in m/s). The values of all experiments are interpolated and extrapolated in the (EESC, CO_2_) space. Most of the extrapolated regions are masked with black.


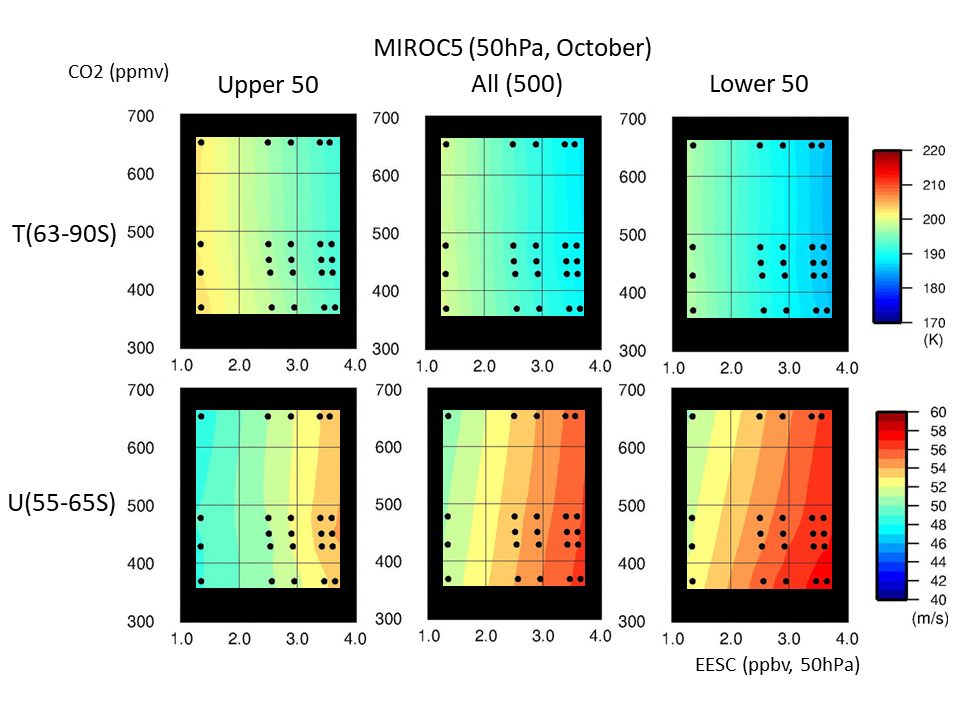


**Supplementary Fig. 12. MIROC5 CCM results for the lower 50 ensemble members, full 500 members and upper 50 members regarding dependence of the polar cap temperature and polar night jet strength in the mid- and high latitudes of the SH (50 hPa, October) on ODS and GHG concentrations.** The polar cap temperatures and polar night jet strength were calculated using the October mean values for daily temperature (63–90°S, 50 hPa) and daily zonal mean zonal wind speed (55–65°S, 50 hPa).


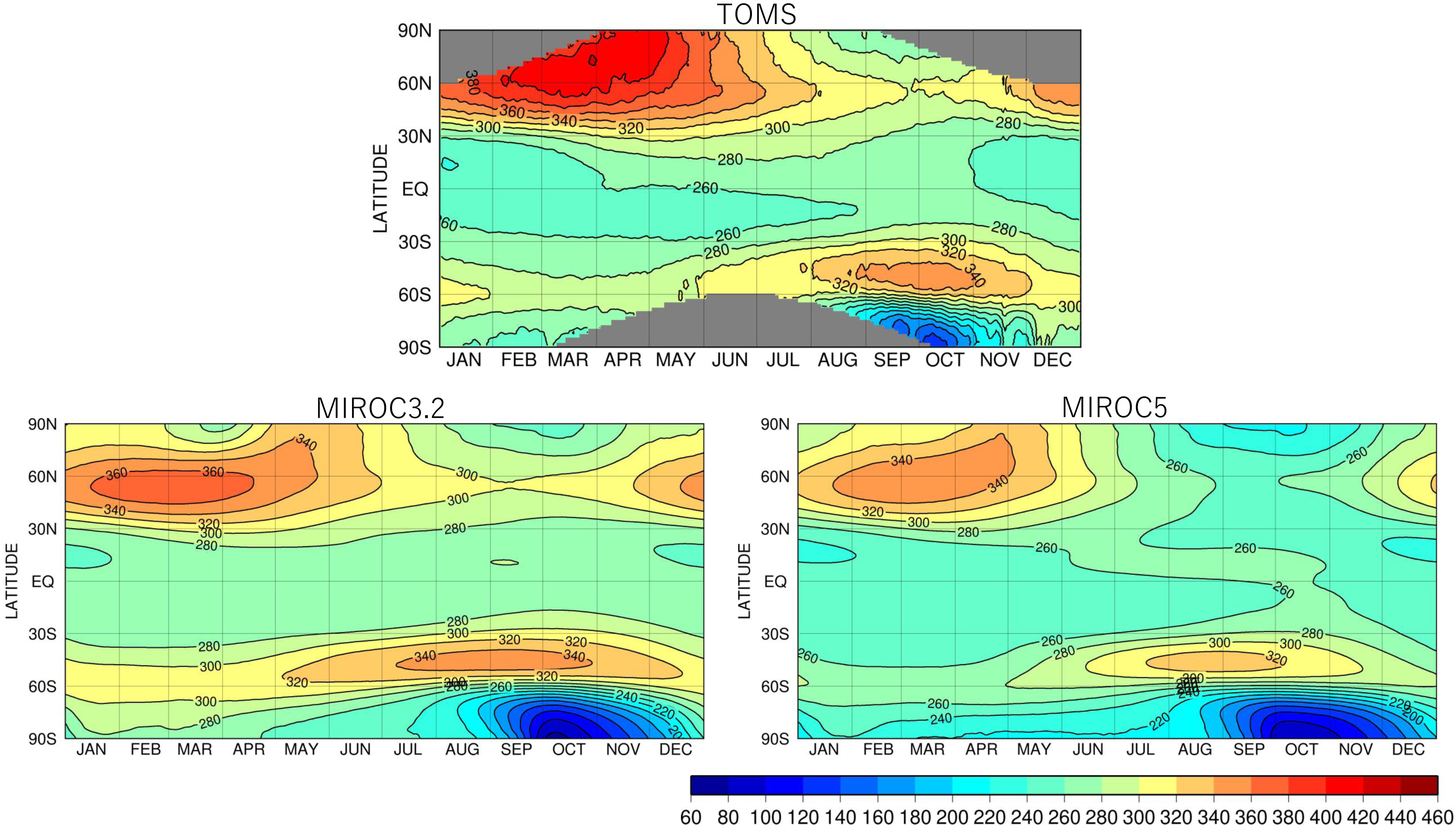


**Supplementary Fig. 13. Time–latitude cross sections of zonal mean column ozone distribution around the year 2000 according to TOMS observation, MIROC3.2 CCM and MIROC5 CCM.** The TOMS-observed column ozone (top panel) is the 8-year average for 1997–2004. The values from MIROC3.2 (bottom left) and MIROC5 (bottom right) are 500-member ensemble means from the ODS-2000&GHG-2000 run. Colour levels represent column ozone values in DU. GMT was used for making figures.


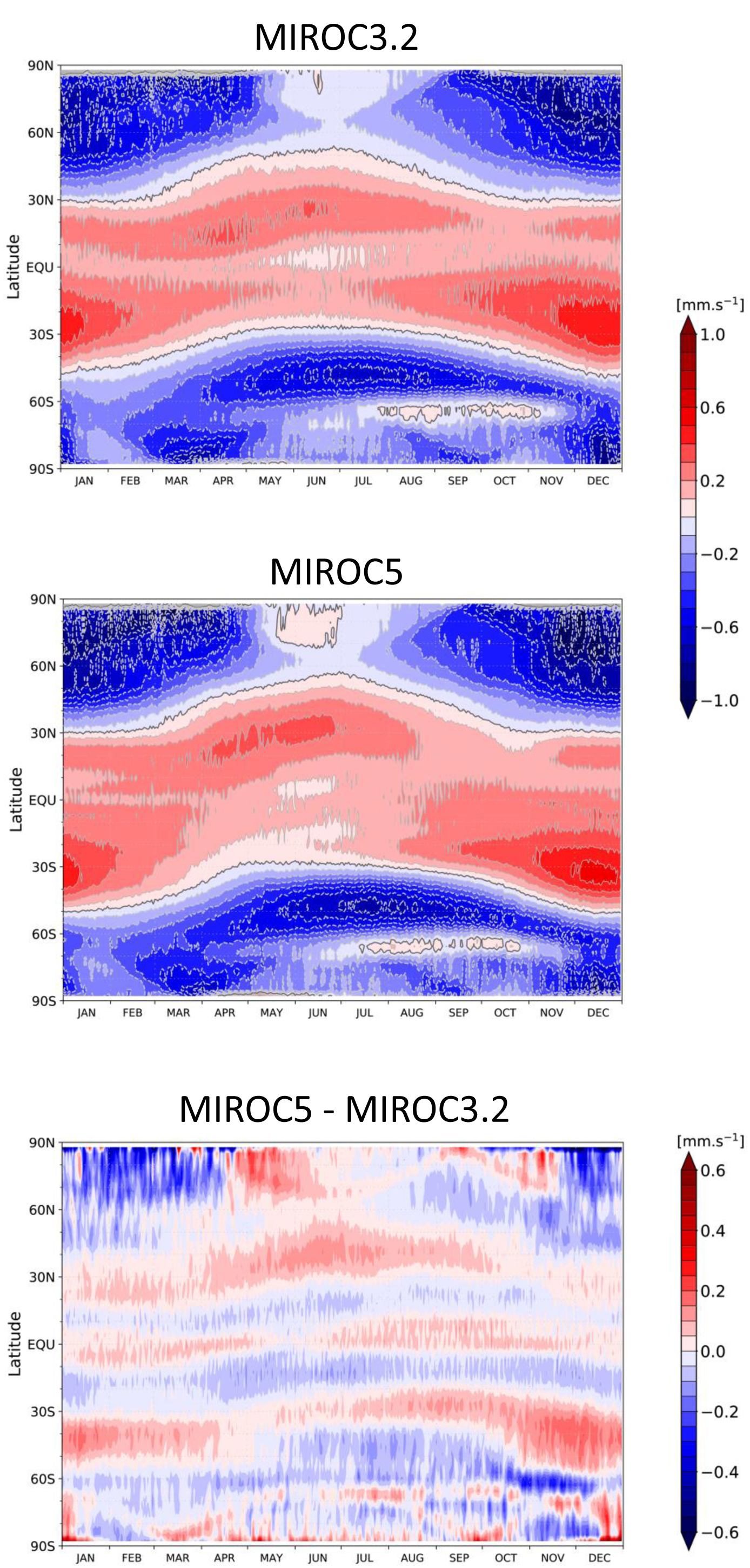


**Supplementary Fig. 14.** **Time–latitude cross sections of the 500-member ensemble mean of the vertical component of the residual mean circulation at 50 hPa.** The top two panels show the ODS-2000&GHG-2000 runs using MIROC3.2 CCM (top) and MIROC5 CCM (middle); the bottom panel shows the difference between them (MIROC5 CCM – MIROC3.2 CCM). Colour levels represent the vertical component of the residual mean circulation in mm/s.
